# High‐Performance Quasi‐Solid‐State Calcium‐Ion Batteries from Redox‐Active Covalent Organic Framework Electrolytes

**DOI:** 10.1002/advs.202512328

**Published:** 2025-11-16

**Authors:** Zhuoyu Yin, Jixin Wu, Ye Tian, Yufei Yuan, Muhua Gu, Lei Cheng, Yanming Wang, Yoonseob Kim

**Affiliations:** ^1^ Department of Chemical and Biological Engineering The Hong Kong University of Science and Technology Clear Water Bay Kowloon Hong Kong SAR 999077 China; ^2^ University of Michigan ‐ Shanghai Jiao Tong University Joint Institute Shanghai Jiao Tong University Shanghai 200240 P. R. China; ^3^ Global Institute of Future Technology Shanghai Jiao Tong University Shanghai 200240 P. R. China; ^4^ Energy Institute The Hong Kong University of Science and Technology Hong Kong, SAR 999077 China

**Keywords:** calcium ion batteries, covalent organic frameworks, quasi‐solid‐state electrolytes, redox activity

## Abstract

Calcium ion batteries (CIBs) are promising for energy storage with volumetric capacity and reduction potential comparable to lithium, while richer in earth abundance. However, sluggish cation transport and unstable cycling performance primarily due to anode surface passivation remain vital challenges for realizing high‐performance CIBs. Herein, two kinds of redox covalent organic frameworks (PT‐COFs and PQ‐COFs) with different‐density carbonyl groups are prepared as quasi‐solid‐state electrolytes (QSSEs) to address those challenges. In particular, PT‐COFs exhibit ionic conductivity of 0.46 and 5.05 mS cm^−1^ at room temperature and 80 °C, respectively, and Ca^2+^ transference number of 0.532. Due to the efficient ionic conduction and intrinsic stability of PT‐COFs structure, the prepared full calcium ion cell with PT‐COFs demonstrates the highest reversible specific capacity of 155.9 mAh g^−1^ at 0.15 A g^−1^ (1 C), and stable cycle performance (capacity retention over 74.6% at 1 A g^−1^ after 1000 cycles). This work shows the effectiveness of the redox COFs and their promising potential as SSE for the development of high‐performance CIBs.

## Graphical abstract

Quasi‐solid‐state calcium‐ion batteries (QSSCIBs) employing redox‐active covalent organic frameworks (COFs) based electrolytes are developed. The COFs’ crystalline porous structures, featuring aligned carbonyl groups, enabled high Ca^2^⁺ conductivity, supported by molecular dynamics simulations of the ion transport mechanism. The fabricated QSSCIBs demonstrate competitive capacity and stability over reported gel/polymer‐based counterparts, as validated by post‐cycling analysis.

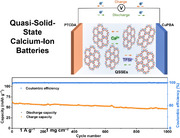

## 1 Introduction 

Rechargeable batteries, represented by lithium‐ion batteries (LIBs), have been enormously effective and convenient in the past 30 years as indispensable electrochemical energy storage devices in portable electronic devices, electric vehicles, etc.^[^ ^1^ ^]^ However, considering limited lithium reserves and sustainability, a market demand for developing alternative batteries, such as the ones utilizing abundant and economical multivalent metal ions, is rapidly increasing.^[^ ^2^, ^3^ ^]^ Among the promising multivalent metals, calcium is especially attractive as it is the fifth earth abundant element, possesses a high theoretically volumetric capacity (2072 mAh cm^−3^), and low redox potential (–2.87 V vs SHE for Ca^2+^/Ca), which is comparable to lithium metal (2046 mAh cm^−3^, –3.04 V vs SHE for Li^+^/Li).^[^ ^4^ ^]^ Although calcium ion batteries (CIBs) are promising as next‐generation energy storage technologies, their development is hindered by sluggish ion conduction and poor cycling performance resulting from the formation of passivation layers.^[^ ^5^, ^6^, ^7^, ^8^ ^]^ Thus, developing suitable electrolytes and electrode materials is urgently needed.

As one of the core components, the electrolyte serves to transport ions and separates the anode and cathode, which critically determines the performance and cycling life of CIBs.^[^ ^9^ ^]^ The mainly developed CIB electrolytes are aqueous ones or liquid electrolytes widely used in LIBs. The aqueous electrolyte uses water as the solvent and regulates types and concentrations of inorganic calcium salts, such as CaCl_2_,^[^ ^10^ ^]^ Ca(NO_3_)_2_,^[^ ^11^ ^]^ Ca(ClO_4_)_2_,^[^ ^12^ ^]^ etc. Organic liquid electrolyte focuses on exploring types of organic solvents together with highly concentrated calcium salts.^[^ ^13^, ^14^, ^15^ ^]^ Zhao‐Karger's group reported a solution of calcium tetrakis(hexafluoroisopropyloxy)borate (Ca[B(hfip)_4_]_2_) in dimethoxy ethane as electrolyte.^[^ ^16^ ^]^ The prepared 0.25 m electrolyte solution showed a high ionic conductivity of 8.3 mS cm^−1^ and a high oxidative stability up to 4.5 V. However, the authors only demonstrated preliminary Ca|Ca symmetric cell data. Tang and co‐workers prepared a 3.5 m electrolyte solution containing calcium bis(fluorosulfonyl)imide (Ca(FSI)_2_) in mixed carbonate solvents to show a conductivity of 0.3 mS cm^−1^ at room temperature (r.t.).^[^ ^17^ ^]^ The authors made full cells comprising 3,4,9,10‐perylenetetracarboxylic dianhydride (PTCDA) as anode and graphite cathode to show 63.9 mAh g^−1^ specific capacity (84.7% retention) at 0.1 A g^−1^ after 350 cycles.

Two critical issues in CIBs are graphite's low capacity and instability at high current densities and the dissolution of anode materials, e.g., PTCDA, into liquid electrolytes. Developing Prussian blue analogues, metal oxides, and phosphates improved the cathode issues.^[^ ^18^ ^]^ However, the electrode dissolution can ultimately only be solved by installing solid‐state electrolytes (SSEs) that possess high thermal and mechanical stability, resistance to leakage, and moderate ion conductivity. Thanks to those merits, the field of all‐solid‐state lithium‐based batteries has been blooming.^[^ ^19^, ^20^, ^21^ ^]^ However, developing all‐solid‐state CIBs is quite challenging due to sluggish ion transport at the interparticles and interfaces.^[^ ^22^ ^]^ Thus, the quasi‐solid‐state electrolyte (QSSE) is a practical way of mitigating the issues from the all‐solid‐state systems while utilizing the advantageous features of liquid systems. Still, the quasi‐solid‐state systems in CIBs are quite rare. Hosein's group reported gel electrolytes, where ionic liquid and calcium salts are embedded in a polyethylene glycol diacrylate matrix.^[^ ^23^ ^]^ This gel electrolyte showed a relatively lower ionic conductivity of 0.17 mS cm^−1^ at r.t., and the full cell's initial capacity of 140 mAh g^−1^ decreased rapidly to 20 mAh g^−1^ after 20 cycles at a constant current of 40 µA (a rate of C/14). Thus, the development of stably operating solid‐state CIBs remains challenging.^[^ ^24^, ^25^ ^]^

Covalent organic frameworks (COFs) with high crystallinity, designability, chemical stability, and regular pore distribution have been widely used in energy storage, gas separation, catalysts, and ion conduction applications.^[^ ^26^ ^]^ Unlike the amorphous and non‐porous polymers, COFs with rigid and periodic pore structures are beneficial for forming interconnected networks and shortening ion diffusion length for fast ion transport.^[^ ^27^ ^]^ The high designability also permits modifying electrochemically active groups into the frameworks for further improvement of ion conduction.^[^ ^19^, ^28^, ^29^ ^]^ Shi's group reported a type of TB‐COF with C═O and C═N active sites as an aqueous CIB anode.^[^ ^30^ ^]^ The highly ordered pore channels together with abundant redox‐active sites in TB‐COF exhibited a Ca^2+^ diffusion coefficient of 10^−6^ cm^2^ S^−1^, which is over two orders of magnitude higher than that in redox organic molecules and inorganic crystals. Lv and co‐workers prepared a type of PTHAT‐COF with pyrazine and pyridinamine units to maximize the amount of C═N sites.^[^ ^31^ ^]^ The aligned and highly concentrated C═N active sites promoted Ca^2+^ diffusion during the insertion/extraction process in the anode. The assembled aqueous CIBs with PTHAT‐COF anode showed a reversible operation over 10000 cycles. These two examples focused on the synthesized COFs being 2D layered sheets and heteroatom‐containing; thus, electron‐rich COFs can store and interact well with Ca^2+^. Inspired by these examples, we designed and prepared carbonyl‐functionalized COF‐based QSSEs for CIBs, which provided new ideas and promising potential for the development of solid‐state CIBs.

**Scheme 1:**
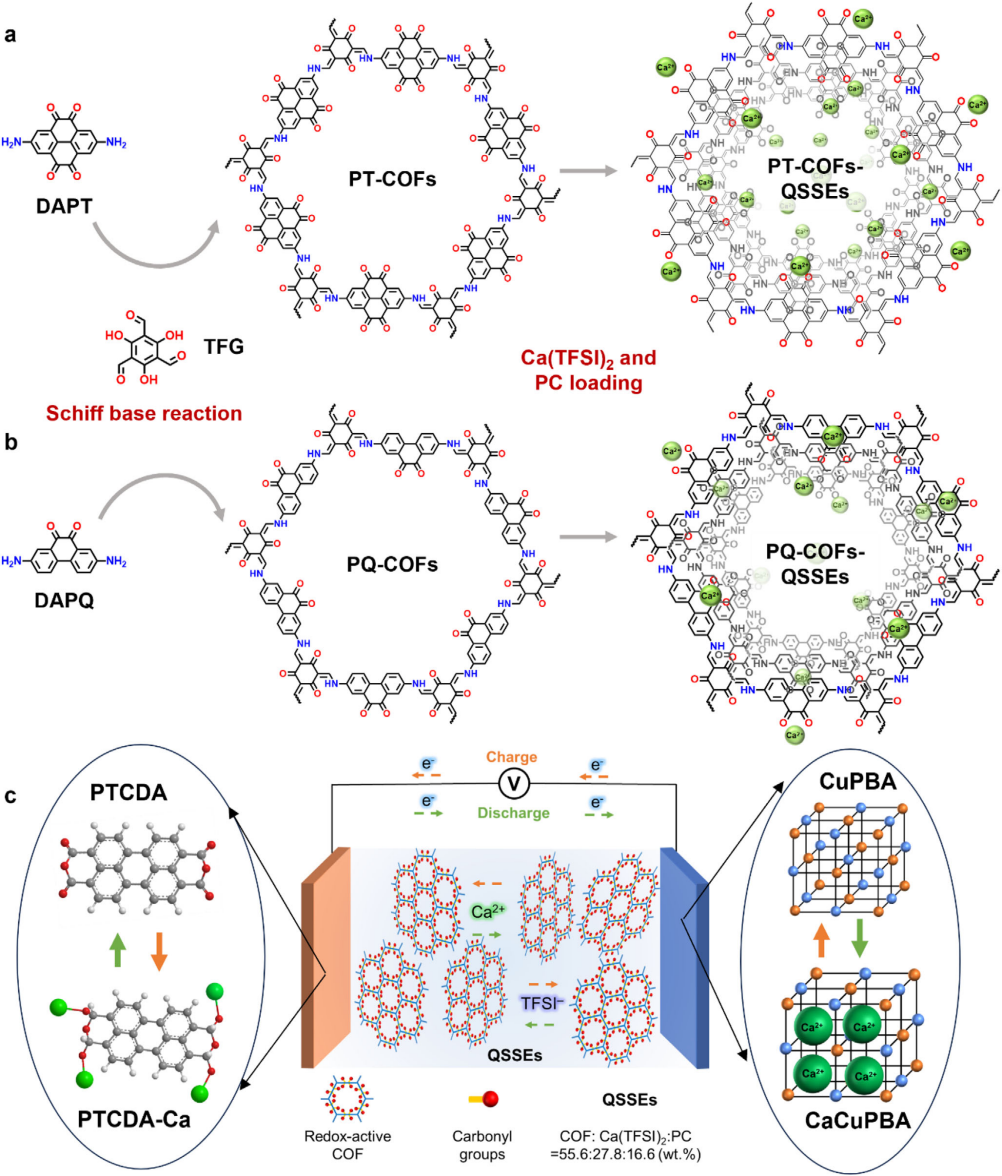
Synthetic processes of making quasi‐solid‐state Ca^2+^‐conductors using pyrene‐tetraone COFs (PT‐COFs) and phenanthrenequinone‐COFs (PQ‐COFs). a,b) First, Schiff‐base condensation reaction is conducted between the monomers: 2,7‐diaminopyrene‐4,5,9,10‐tetraone (DAPT) and triformylphloroglucinol (TFG) for PT‐COFs and 2,7‐diamino‐9,10‐phenanthrenequinone (DAPQ) and TFG for PQ‐COFs, followed by loading of Ca(TFSI)_2_ and PC. c) Schematic showing working full cell QSSEs CIB based on COF electrolytes.

To explain the overall scope of the work briefly, we synthesized two types of redox‐active COFs, pyrene‐tetraone COFs (PT‐COFs) and phenanthrenequinone‐COFs (PQ‐COFs), having different densities of carbonyl groups (**Scheme** **1**) as electrolyte matrices; then added calcium(II) bis(trifluoromethanesulfonimide) (Ca(TFSI)_2_) salt and propylene carbonate (PC) to prepare QSSEs for CIBs. The COFs, Ca(TFSI)_2_, and PC ratio are 55.6:27.8:16.6 (wt.%). Hereafter, such electrolyte systems are called PT‐COFs‐QSSEs and PQ‐COFs‐QSSEs, respectively. The PT‐COFs‐QSSEs showed an ionic conductivity of 0.46 and 5.05 mS cm^−1^ at r.t. and 80 °C, respectively. Using PTCDA as anode and Cu‐coordinated Prussian blue analogue (CuPBA) as cathode,^[^ ^32^ ^]^ the full CIB cells with PT‐COFs‐QSSEs exhibited a maximum capacity of 155 mAh g^−1^ at 0.15 A g^−1^ (1 C = 150 mAh g^−1^, corresponding to the current density for full discharging process in 1 h), and stably operated over 1000 cycles at high current density of 1 A g^−1^ (6.7 C). This is the first demonstration to show the feasibility of achieving stable and durable CIBs based on redox COF electrolytes at such high current density.

## 2 Results and Discussion

**Figure 1:**
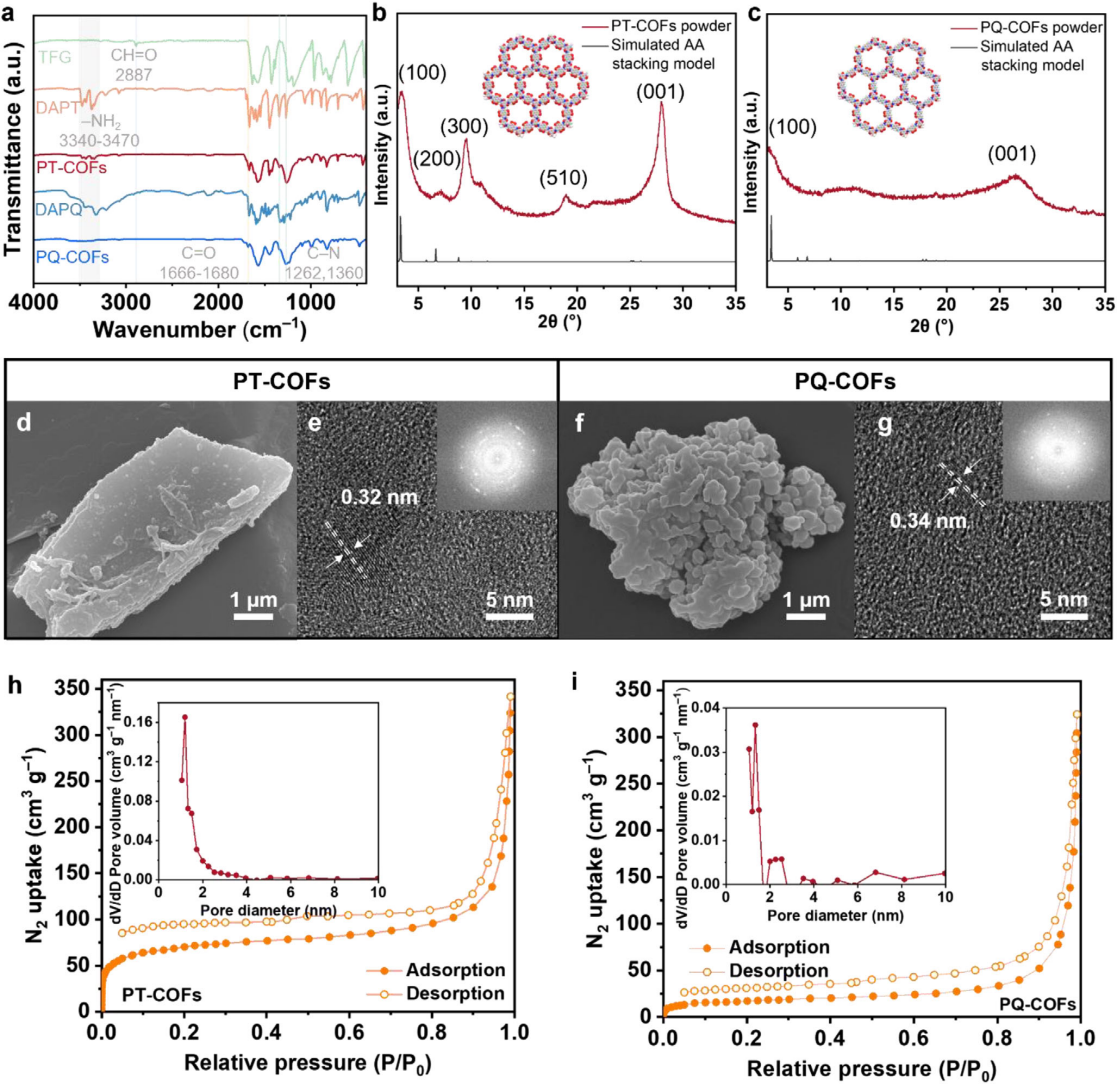
Physical characterization of PT‐COFs and PQ‐COFs. a) Fourier‐transform infrared spectroscopy (FTIR) of the synthesized COFs and their starting monomers. b,c) Powder X‐ray Diffraction (PXRD) patterns and simulated stacking models of PT‐ and PQ‐COFs, respectively. d–g) Scanning electron microscope (SEM) and transmission electron microscope (TEM) images of PT‐ and PQ‐COFs, respectively. For TEM images, insets are the corresponding fast Fourier transform patterns. h,i) N_2_ adsorption/desorption isotherms of PT‐ and PQ‐COFs, respectively. Insets are pore size distribution data.

We synthesized the PT‐COFs and PQ‐COFs using the solvothermal method through a Schiff base reaction, involving mesitylene, 1,4‐dioxane, and acetic acid, between 2,7‐diaminopyrene‐4,5,9,10‐tetraone (DAPT) or 2,7‐diamino‐9,10‐phenanthrenequinone (DAPQ) with 2,4,6‐triformylphloroglucinol (TFG), respectively (Scheme 1; See Supporting Information for synthetic details).^[^ ^33^, ^34^, ^35^ ^]^ The successful synthesis was confirmed by extensive characterization methods. First, the ^1^H Nuclear Magnetic Resonance spectroscopy (NMR) showed the successful preparation of DAPT and DAPQ monomers (Schemes S1 and S2 and Figure S1–S5, Supporting Information). Fourier‐transform infrared spectroscopy (FTIR) revealed the formation of backbones (**Figure** **1**a). The characteristic N─H bond stretch, 3340–3470 cm^−1^, from amino groups in DAPT and DAPQ appeared as a weak to medium intensity, somewhat broad peak from the COF products. The C═O stretching vibration of aldehyde groups at 2887 cm^−1^, in TFG, disappeared in the COF products after reacting with amino monomers. Due to the keto‐enol tautomerization after the imine condensation, both COF products exhibited highly intense C─N vibration peaks of aliphatic amine structure at 1262–1271 cm^−1^ and decreased intensity of C─N vibration at 1360 cm^−1^ from aromatic amine structure. Powder X‐ray diffraction (PXRD) patterns showed that both samples are highly crystalline (Figure 1b,c). The characteristic peaks at 3.4° for PT‐COFs and 3.2° in PQ‐COFs were assigned to their (100) lattice plane, while the high‐degree broad peaks of 27.9° and 26.9° corresponded to their (001) plane, indicating their π‐π stacking structure. Additionally, the PT‐COFs also showed peaks at 6.8°, 10.2°, and 18.9°, corresponding to (200), (300), and (510) lattice planes, respectively. Both COF products exhibited an AA stacking arrangement.

Scanning electron microscopy (SEM) images showed that PT‐COFs look like nanosheets while the PQ‐COFs are more like particle aggregates (Figure 1d,f). The transmission electron microscopy (TEM) analysis also showed that both COFs had clear lattice fringes with a mean spacing of 0.32 and 0.34 nm, respectively (Figure 1e,g). Due to the high magnification of TEM images and high crystallinity, the fast Fourier transform images of both COFs exhibited strong electron diffraction patterns. The surface area and pore size distribution of the prepared COFs were measured through N_2_ adsorption/desorption in a liquid nitrogen atmosphere. PT‐COFs and PQ‐COFs exhibited Type II isotherms with N_2_ uptake of 341 and 324 cm^3^ g^−1^, and Brunauer–Emmett–Teller surface areas of 192 and 178 m^2^ g^−1^, respectively (Figure 1h,i). The Barrett‐Joyner‐Halenda pore size distribution analysis showed the PT‐COFs and PQ‐COFs had an average pore diameter of up to 1.3 and 1.7 nm, respectively. The thermal stability of prepared COFs was evaluated by thermal gravity analysis from 25 to 800 °C with a heating rate of 10 °C min^−1^. Both exhibited one degraded stage during the heating process. PT‐COFs and PQ‐COFs were quite stable: more than 85 wt.% remained up to 413 and 275 °C, respectively (Figure S6, Supporting Information). The higher‐density carbonyl groups in the framework were beneficial for forming larger π‐conjugated structures and increasing the local polarity, contributing to the higher thermal stability of PT‐COFs and conduction of multivalent ions.

### 2.1 Electrochemical Properties

**Figure 2:**
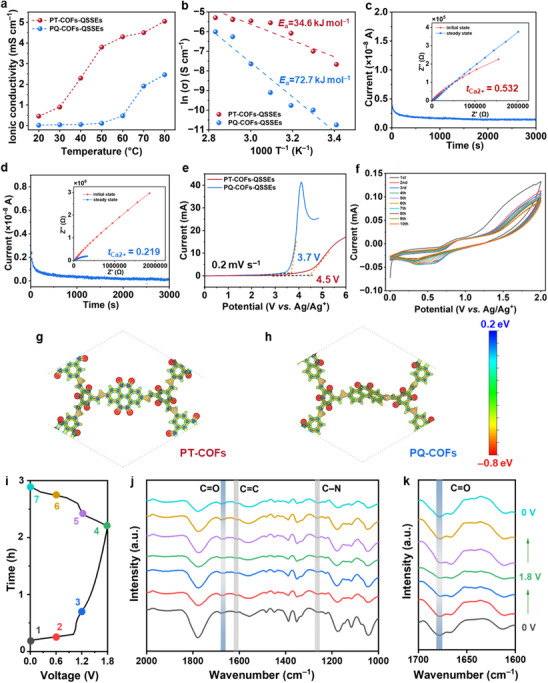
Electrochemical evaluation of PT‐COFs and PQ‐COFs. a) Ionic conductivity of PT‐COFs‐QSSEs and PQ‐COFs‐QSSEs at different temperatures. b) Arrhenius plots of ionic conductivity as a function of temperature for both types of QSSEs. c,d) Ca^2+^ transference number (*t*_Ca2+_) of PT‐ and PQ‐COFs‐QSSEs calculated using Bruce−Vincent−Evans technique in Ca|QSSEs|Ca symmetric cell at r.t., respectively. e) LSV curves of PT‐COFs‐QSSEs and PQ‐COFs‐QSSEs at a scan rate of 0.2 mV s^−1^ in a three‐electrode system with Pt as working and counter electrode, and Ag/Ag^+^ as reference electrode. f) Cyclic voltammetry (CV) curves of PT‐COFs at a scan rate of 0.2 mV s^−1^ with the first ten cycles. g,h) Molecular electrostatic potentials (MEP) of the core repeating units of PT‐COFs and PQ‐COFs, respectively. i–k) Ex situ FTIR spectra of PT‐COFs and the corresponding charge‐discharge profiles.

Ionic conductivity is one of the critical factors in evaluating the practical feasibility of electrolytes, especially in solid‐state electrolytes for multivalent ion batteries, because of the intrinsic diffusion of such ions. The higher ionic conductivity of electrolytes can improve the ion transfer efficiency, enabling fast charge/discharge processes. For this purpose, we made optimized composite SSEs consisting of PT‐ or PQ‐COFs, Ca(TFSI)_2_, and PC in the ratio of 55.6:27.8:16.6 (wt.%). Hereafter, the composite mixture samples are denoted as PT‐ or PQ‐COFs‐QSSEs (See Table S1, Supporting Information for control samples and their conductivity values), and they retained solid‐state structure integrity after standing for 24 h (Figure S7a, Supporting Information). As our CIBs are not with a calcium metal anode, which could supply Ca^2+^, we needed to add Ca^2+^ source; thus we added Ca(TFSI)_2_. Also, we found that electrolytes without liquid organic solvents, such as PC or dimethoxyethane, are barely conductive (1.4 ×10^−6^ mS cm^−1^, Table S1, Supporting Information). While all‐solid‐state cells could have been realized under applied operational pressure, we deliberately opted to assemble and test the cells without external pressure to better align with practical battery manufacturing conditions and application scenarios. In both prepared QSSEs, ionic conductivities increased, and the ionic‐conducting impedance decreased along with temperature, indicating the ionic transfer process exhibited thermodynamically driven features. Specifically, the PT‐COFs‐QSSEs exhibited a conductivity of 0.46 and 5.05 mS cm^−1^ at r.t. and 80 °C, respectively, while PQ‐COFs‐QSSEs showed 0.021 and 2.46 mS cm^−1^ at r.t. and 80 °C, respectively (**Figure** **2**a). The higher ionic conductivity of PT‐COFs‐QSSEs stemmed from the fact that they had more carbonyl groups to provide abundant conduction moieties and shorter transport pathways among the moieties – PT‐ and PQ‐COFs have 24 and 12 carbonyl groups in the hexagonal repeating units, respectively. This is consistent with the lower activation energy in PT‐COFs‐QSSEs (34.6 kJ mol^−1^) than that of PQ‐COFs‐QSSEs (72.7 kJ mol^−1^) (Figure 2b). The corresponding Nyquist plots exhibited semicircle patterns at a high‐frequency range (10^6^–10^3^ Hz) and one sloping line at a low‐frequency range (10^3^–10^0^ Hz), representing the interphase charge‐transfer resistance between QSSEs and electrodes and ion‐conducting impedance, respectively (Figure S7b,c, Supporting Information). Compared to PQ‐COFs‐QSSEs, PT‐COFs‐QSSEs consistently showed smaller ion‐conducting impedance under the same conditions. This smaller impedance indicated faster ion diffusion and transport,^[^ ^36^, ^37^, ^38^ ^]^ which corresponded to the higher ion conductivity and lower activation energy of PT‐COFs‐QSSEs. The PT‐ and PQ‐COFs‐QSSEs exhibited Ca^2+^ transference (*t*_Ca2+_) numbers of 0.532 and 0.219, respectively (Figure 2c,d). It was logical to have high *t*_Ca2+_ from carbonyl‐rich PT‐COFs‐QSSEs.

Electrochemical stable windows (ESWs) should be identified to understand the safe working potential ranges of electrolytes. Thus, linear sweep voltammetry (LSV) experiments were conducted to evaluate the ESWs of the prepared QSSEs in a three‐electrode system with Pt as both working electrode and counter electrode, and Ag/Ag^+^ as reference electrode (Figure 2e). When tested at a scan rate of 0.2 mV s^−1^, PT‐ and PQ‐COFs‐QSSEs exhibited electrochemical oxidation stability at 4.5  and 3.7 V, respectively. The better oxidation stability of PT‐COFs‐QSSEs was attributed to the larger conjugation structure. The highest occupied molecular orbital (HOMO) and lowest unoccupied molecular orbital (LUMO) values were calculated to support this phenomenon. The gap turned out to be 2.22  and 2.19 eV for the DAPT and DAPQ monomers, respectively (Figure S8, Supporting Information), and the higher HOMO–LUMO gap indicated the better stability in PT‐COFs‐QSSEs. The reductive stability of PT‐/PQ‐COFs‐QSSEs was also evaluated under the same conditions. Especially, the PT‐/PQ‐COFs‐QSSEs exhibited electrochemical reductive stability at –3.9  and –3.6 V (Figure S9, Supporting Information). Subsequently, we tested redoxactivities of the PT‐COFs using cyclic voltammetry (CV) method, where PT‐COFs, activated carbon, Ag/Ag^+^ and 0.5 m Ca(TFSI)_2_ in propylene carbonate are used as working electrode, counter electrode, reference electrode, and electrolyte, respectively. The CV curves with the first ten cycles at a scan rate of 0.2 mV s^−1^ exhibited an oxidation peak at 0.9 V and a reduction peak at 0.3 V, corresponding to the reversible coordination of Ca^2+^ by C═O groups in the PT‐COFs (Figure 2f). The fact that the ten cycling curves overlapped nearly perfectly indicated the excellent reversibility of Ca^2+^ coordination processes and stability of the PT‐COFs. The electrochemical kinetics for PT‐COFs were explored by CV profiles at various scan rates, 0.5, 1, 2, 3, 5, and 10 mV s^−1^ (Figure S10a, Supporting Information). The relationship between redox peak current (*i*) and scan rates (*v*) can be calculated by the formula of *i* = a*v* ^b^, where *a* and *b* are the corresponding parameters, and *b* = 0.5 indicates the diffusion‐controlled process and *b* = 1 represents the surface‐controlled capacitive process.^[^ ^39^, ^40^ ^]^ The logscale relationship between peak current and scan rate of the PT‐COFs showed that PT‐COFs have both diffusion‐ (*b* of 0.95 for anodic peaks) and capacitive‐controlled (*b* of 0.61 for cathodic peaks) reactions (Figure S10b, Supporting Information).

To visualize the molecular recognition sites on the COFs for Ca^2+^ coordination and transport, we conducted molecular electrostatic potential (MEP) studies.^[^ ^31^ ^]^ The MEP of the repeating units in both COFs showed more negative potentials concentrated on the carbonyl oxygen atoms in both COF units, indicating that these carbonyl groups are responsible for bonding with electrophilic cations (Figure 2g,h). To further explore the reversible Ca^2+^ coordination process with the carbonyl groups in the PT‐COFs, we conducted ex situ FTIR during the courses of charging from 0 to 1.8 V and discharging from 1.8 to 0 V over 3 h (Figure 2i). We observed two clear carbonyl peaks at 1670 and 1786 cm^−1^ from pristine samples, **1**, (Figure 2j; 2k for 1600–1700 cm^−1^ magnified image). The characteristic peak at 1786 cm^−1^ was attributed to the C═O stretching vibration from the PC additives. In contrast, the peak at 1670 cm^−1^ was assigned to the ketone‐type C═O vibration from PT‐COFs, an assignment supported by its conjugated structure. Given that conjugated carbonyl groups generally exhibit enhanced redox activity due to improved electron delocalization, we used the peak at 1670 cm^−1^ as an indicator to track the redox process during electrochemical cycling. As the charging progressed from **1** to **4**, the peak intensity noticeably decreased, indicating a reduction occurred to coordinate Ca^2+^. From **4** to **7**, the reverse process recovered the peak, indicating Ca^2+^ de‐coordination occurred. These data sets corroborate that the carbonyl groups work as electrochemically active sites for Ca^2+^ transport.

**Figure 3:**
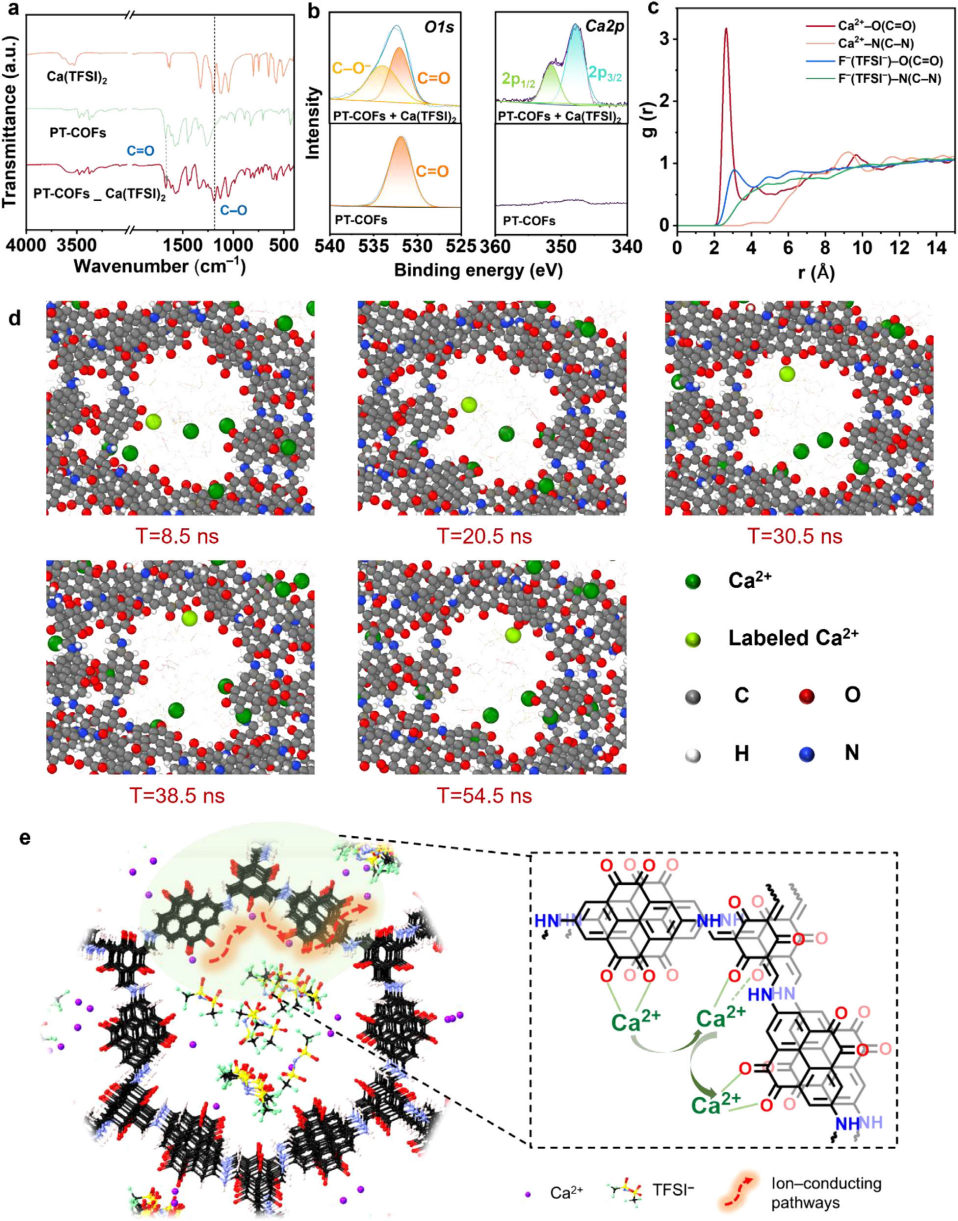
Composites characterization and molecular dynamic simulation of Ca(TFSI)_2_ in PT‐COFs. a) FTIR spectrum of PT‐COFs, Ca(TFSI)_2_ and their composite. b) XPS spectra of PT‐COFs and the composites. c) Radial distribution functions (RDF) of Ca^2+^ and TFSI^–^ ions in relation to redox centers (O, N atoms) in PT‐COFs. d) Simulation trajectory frame at 8.5, 20.5, 30.5, 38.5, and 54.5 ns of PT‐COFs‐QSSEs box to present Ca^2+^ transport mechanism. e) Illustration of ion‐conducting pathways in PT‐COFs‐QSSEs (Black: C; Red: O; Blue: N; White: H).

Additionally, FTIR and X‐ray photoelectron spectrometry (XPS) spectra were employed to confirm the interaction between carbonyl groups from PT‐/PQ‐COFs and Ca(TFSI)_2_. Take PT‐COFs as example, after the incorporation of Ca(TFSI)_2_ in PT‐COFs, the blue shift of C═O bond vibration peak from 1668 to 1664 cm^−1^ and the appearance of new peak at 1188 cm^−1^ from C─O vibration indicated the strong interaction between PT‐COFs and Ca^2+^ (**Figure** **3**a). Moreover, the O1s of XPS spectra also showed an increased peak at binding energy of 532.4 eV in the composite of PT‐COFs and Ca(TFSI)_2_, which was attributed to the C─O bond and consistent with the FTIR results (Figure 3b). The similar blue shift of C═O vibration peak and new C─O peaks appearance were also observed in the composite of PQ‐COFs and Ca(TFSI)_2_ (Figure S11a,b, Supporting Information). Therefore, the C = O groups on the PT‐/PQ‐COFs backbone were confirmed as active sites for Ca^2+^ binding and transport.

To further reveal the Ca^2+^ transport behaviors at the molecular scale, we performed molecular dynamics (MD) simulations for PT‐ and PQ‐COFs‐QSSEs systems. The model was constructed with the ratio of PT‐/PQ‐COFs, PC, and Ca(TFSI)_2_ that was consistent with the prepared QSSEs. All molecular dynamics simulations were performed using a large atomic molecular massively parallel simulator (LAMMPS).^[^ ^41^ ^]^ The initial configurations and force field parameters were prepared in MedeA.^[^ ^42^ ^]^ The interactions at all‐atom simulation are described by using a Polymer Consistent Force‐Field (PCFF+).^[^ ^43^, ^44^ ^]^ This force field has been successfully applied to the description of polymer electrolyte simulations.^[^ ^45^, ^46^ ^]^ Within this model, electrostatics are characterized as a point‐charge model. This results in an overestimation of the interionic forces in the electrolyte. We partially mitigate it by employing a common approach in classical molecular dynamics simulations: scaling the partial charges of ions by a 0.7 factor.^[^ ^47^, ^48^, ^49^ ^]^ All atomic charges in the simulation can be found in Table S2 (Supporting Information). However, under the same electrolyte concentration as experiments, the ionic diffusion conductivity will still be underestimated. Therefore, we perform structural relaxation and MD simulations at elevated temperatures, which is also a commonly used method to accelerate the understanding of ion transport properties. The relaxation process to reach the target temperature of 473 K and pressure of 1 atm includes a series of energy minimization and dynamic processes under canonical (nVT) and isothermal−isobaric (nPT) statistical ensemble, with a total duration of 10 ns. Following that, we yield well‐relaxed models with stable density, volume, and energy levels. More details of our configurations and calculations can be found in the Supporting Information.

Radial distribution functions (RDF) calculations centered on Ca^2+^ and TFSI^–^ were conducted to investigate the binding interactions between electrolyte ions and functional groups in both PT‐COFs and PQ‐COFs (Figure 3c; Figure S11c, Supporting Information). It is worth emphasizing that the configuration of the first peak plays a crucial role in ion migration. Compared to Ca^2+^–N (nitrogen from amino group) interactions, Ca^2+^–O interaction (oxygen from carbonyl groups) showed a characteristic peak at ≈ 2.5 Å and higher intensity, suggesting that the carbonyl groups are prior redox‐active sites for Ca^2+^ trapping in both COFs. Moreover, different from disordered and weaker intensity of F─O bonds (fluorine from TFSI^–^), the sharp characteristic peaks of Ca^2+^–O interaction are presented in both COFs models, indicating that the carbonyl groups are the preferred sites for Ca^2+^ ions coordination, which is consistent with the MEP suggestion and ex situ FTIR results. This difference also suggests that Ca^2+^ and TFSI^–^ in the electrolyte within the composite system exhibited a dissociation tendency. Based on the relaxed configuration (S7.2, Supporting Information), we further extended the MD simulation time. We conducted a 50 ns simulation under NPT ensemble on the fully optimized structure to capture the migration behavior of Ca^2+^ in the PT‐COFs‐QSSEs system and display the transport mechanism (Figure S12a, Supporting Information). Especially, one light green ball labelled Ca^2+^ was selected to visualize the transport pathways. The Ca^2+^ was observed hopping between carbonyl groups in the PT‐COFs channels during the simulation. From the frame of time = 8.5 ns to time = 30.5 ns, we can observe that the labelled Ca^2+^ left the original carbonyl binding site and moved to the adjacent carbonyl oxygen binding site. Later, from 30.5 ns to 54.5 ns, it again left adjacent carbonyl oxygen atoms and migrated to other carbonyl groups within the channel. (Figure 3d; Figure S12c and Video S1, Supporting Information). Additionally, we used the trajectory calibration function in ovito(visualization software)^[^ ^50^ ^]^ to draw Ca^2+^ movement trajectories from 8.5 to 54.5 ns and colored them with unwrapped position in the ***z*** direction. It was clear that Ca^2+^ not only hopped within the ***x–y*** plane but also transported along the ***z*** direction (Figure S12c–f, Supporting Information). Combined with above studies, we proposed that the carbonyl groups serve as binding sites, suggesting a mechanism that facilitates Ca^2+^ hopping within the COF channels (Figure 3e). Compared to the DAPQ unit, the DAPT unit with more and higher‐density carbonyl groups can form shorter transfer intervals and more continuous conduction pathways, which is beneficial for reducing the ion transfer energy barrier for fast ion transport.

The electronic conductivity of PT‐/PQ‐COFs was also evaluated by the four‐point probe (van der Pauw) method at r.t. with an average pellet thickness of 30 µm. The PT‐COFs and PQ‐COFs exhibited electronic conductivity of 7.02 × 10^−6^ and 4.94 × 10^−6^ S cm^−1^ at r.t., respectively (Figure S13a, Supporting Information). DFT band structure calculations displayed that bandgaps of PT‐COFs and PQ‐COFs were close to zero, indicating their good intrinsic electronic conductivity for redox reaction, which was beneficial to faster Ca^2+^ binding and transport (Figure S13c,d and Tables S3 and S4, Supporting Information).

**Figure 4:**
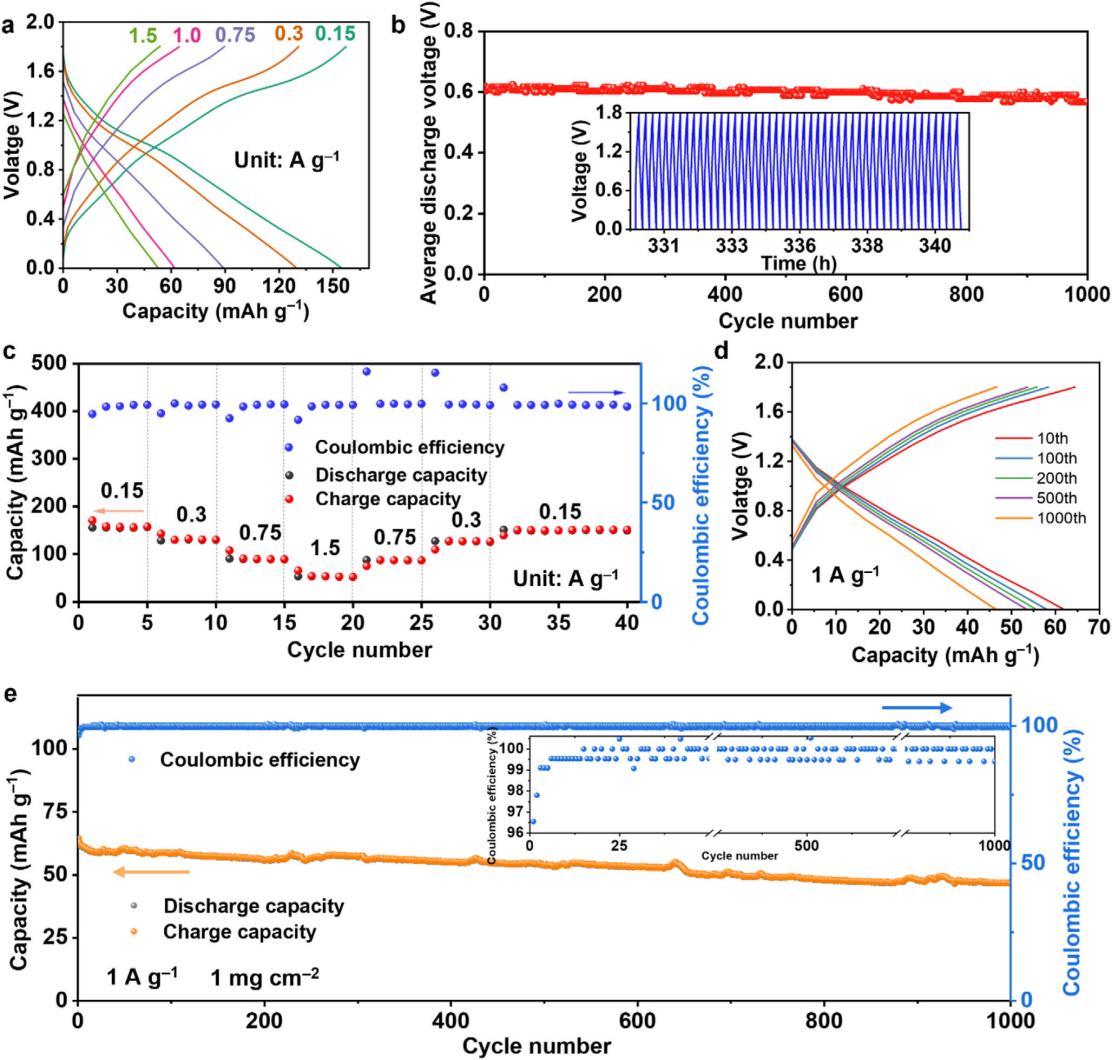
Electrochemical performance of PTCDA|PT‐COFs‐QSSEs|CuPBA coin cells. a) Charge/discharge curves at various current densities. b) Average discharge voltage up to 1000 cycles. Inset shows discharge voltage ranges for the last 40 cycles. c) Charge/discharge capacities upon changing rates and their corresponding Coulombic efficiency. d) Selected charge/discharge curves from 10th, 100th, 200th, 500th, and 1 000th cycles at 1 A g^−1^. e) Long‐term operation of the full cell at 1 A g^−1^. The inset shows the coulombic efficiency at different cycles in detailed y‐axis scales. Comparative full cell data of PTCDA|PQ‐COFs‐QSSEs|CuPBA and PTCDA|0.5 m Ca(TFSI)_2_ in PC|CuPBA are presented in Figures S16 and S17 (Supporting Information), respectively.

With the understanding of our QSSEs, we moved on to fabricating CIB cells. First, we identified anode and cathode materials as suitable electrode materials that fit with electrolytes’ ESW can make a performance–cost balance for long‐term stable operations. For the anode, we chose PTCDA, an electroactive organic molecule with low cost and high stability, as it demonstrated promising potential as an anode material to host multivalent ions.^[^ ^51^ ^]^ PTCDA molecules with four carbonyl groups can deliver a two‐electron process for Ca^2+^ insertion/de‐insertion. For the cathode, we chose CuPBA as it is easy to prepare, cheap, and can reversibly intercalate multivalent alkaline ions.^[^ ^52^ ^]^ The successful synthesis of CuPBA was characterized by PXRD and SEM (Figure S14, Supporting Information). The working voltage ranges of both electrodes were evaluated by CV profiles, showing anodic and cathodic peaks at –0.59/–0.88 V  and 0.76/0.63 V, respectively (Figure S15, Supporting Information). These range fitted with our COF‐based QSSEs and showed promise for the reversible reaction of ion insertion and de‐insertion. Utilizing those materials with the prepared COF‐based QSSEs, we fabricated coin cells (Scheme 1c). The assembled PTCDA|PT‐COFs‐QSSEs|CuPBA full cell showed the galvanostatic charge/discharge profiles when cycled at a voltage range of 0–1.8 V (**Figure** **4**a). It presented discharge capacities of 155.9 and 62.2 mAh g^−1^ at current densities of 0.15 (1 C) and 1.0 A g^−1^, respectively (Table S5, Supporting Information). The corresponding volumetric energy density and gravimetric energy density of PTCDA|PT‐COFs‐QSSEs|CuPBA full cell were calculated as 170.7 Wh L^−1^ and 32.9 Wh kg^−1^ at 1 C based on the entire coin cell (2032), respectively. For comparison, the full cell with PQ‐COFs‐QSSEs showed significantly lower discharge capacities of 59.6 and 13.5 mAh g^−1^ at 0.03 and 0.3 A g^−1^, respectively (Figure S16a,b and Table S6, Supporting Information). The full cells with PT‐COFs‐QSSEs exhibited an average discharge voltage of 0.62 V up to 1000 cycles (Figure 4b) and kept stable in the entire testing period. We further tested the cells at different current densities. Starting from 0.15 A g^−1^, we increased the current densities up to 1.5 A g^−1^, then lowered it back down to 0.15 A g^−1^ (Figure 4c). The discharging capacities recorded 155.9, 130.9, 89.6, and 53.3 mAh g^−1^ for current densities of 0.15, 0.3, 0.75, and 1.5 A g^−1^, respectively. When the current density was lowered back down to 0.15 A g^−1^, the cell showed high stability to record a discharging capacity of 149.6 mAh g^−1^, showing 96% of the initial capacity was retained. While the cell with PQ‐COFs‐QSSEs showed discharge capacities of 60.3, 49.2, 35.7, and 14.3 mAh g^−1^ for current densities of 0.03, 0.06, 0.1, and 0.3 A g^−1^, respectively (Figure S16c, Supporting Information).

The full cell with PT‐COFs‐QSSEs showed excellent performance during the 1000 charging/discharging cycles at 1 A g^−1^ and cathode loading of 1 mg cm^−2^ (Figure 4e). The charge/discharge curves were nearly the same, demonstrating their excellent reversibility and negligible polarization. This full cell exhibited 50.3 mAh g^−1^ discharge capacity, 80.9% of the initial capacity, after 700 cycles, and 46.4 mAh g^−1^ discharge capacity, 74.6% of the initial capacity, after 1000 cycles tested at 1 A g^−1^ (Figure 4d). Although the specific capacity of our QSSEs‐based CIBs didn't exceed that of the best‐reported aqueous CIBs, its impressive performance established a new record among QSSEs‐based CIBs and was highly competitive with other state‐of‐the art CIBs employing polymer or gel electrolytes (Table S7, Supporting Information). As a control sample, the same type of full cell with PQ‐COFs‐QSSEs was prepared and tested. When tested at 0.1 A g^−1^, its specific capacity decreased faster. Only 80%, 60%, and 51% remained after 50, 200, and 500 cycles (Figure S16d, Supporting Information). We prepared another control sample containing purely liquid electrolytes, 0.5 m Ca(TFSI)_2_ in PC. The same type of full cell showed a very low initial capacity of 28 mAh g^−1^ at 0.1 A g^−1^. When further tested, it rapidly decayed to 12 mAh g^−1^ only after 50 cycles (Figure S17, Supporting Information). These extensive studies highlight the PT‐COFs‐QSSEs’ rapid and durable Ca^2+^ conduction as well as suitability for the PTCDA anode and CuPBA cathode.

**Figure 5:**
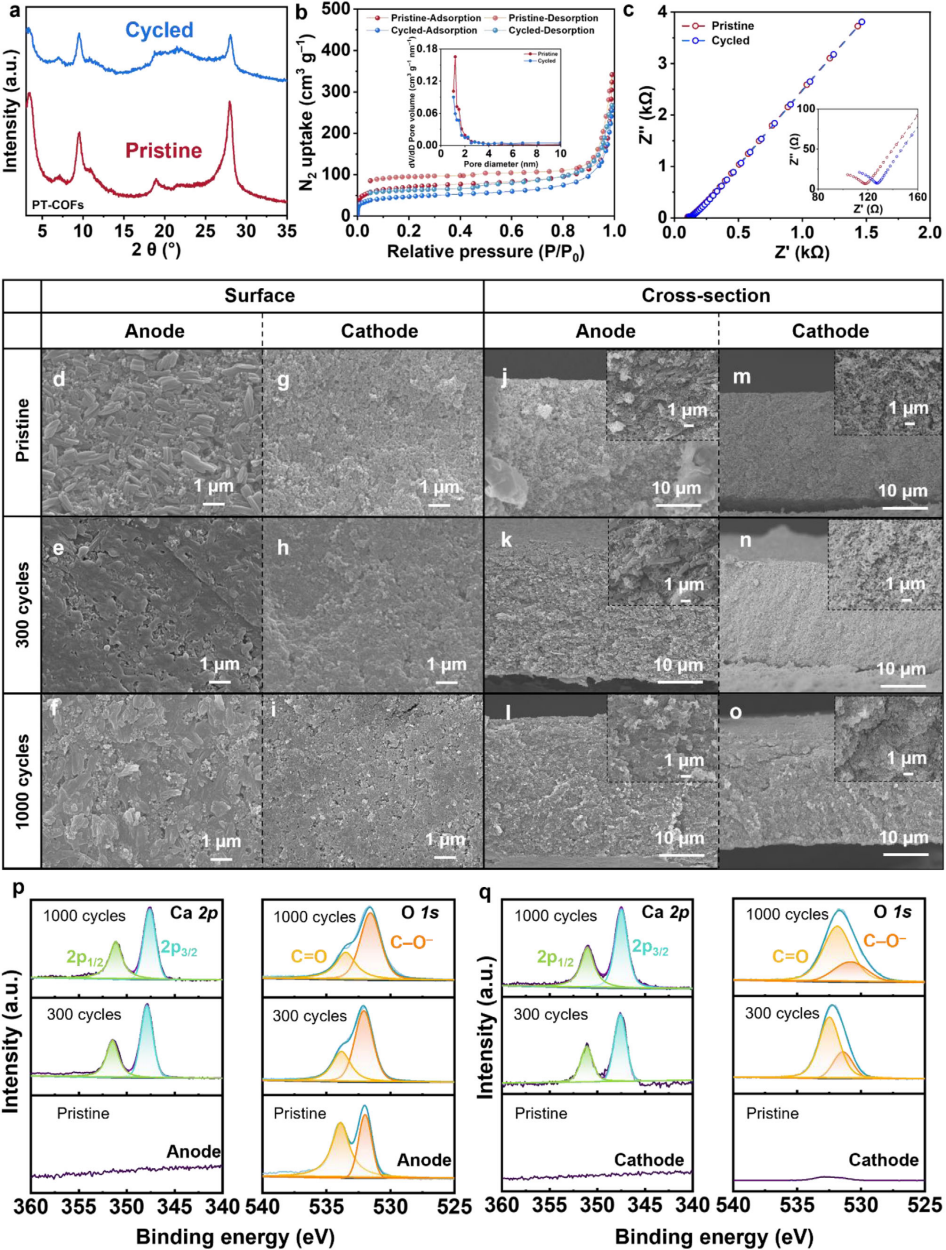
Post‐mortem analysis of PT‐COFs‐QSSEs electrolyte, PTCDA anode, and CuPBA cathode of the full cells. Three data sets, pristine, after 300 cycles at 0.1 A g^−1^, and 1000 cycles at 1 A g^−1^ are compared for all analyses in this figure. After the battery testing at 0.1 A g^−1^ for 300 cycles, we washed PT‐COFs‐QSSEs in methanol for 24 h, filtered the solution to remove the salts and PC, and dried the filtrates at 80 °C to obtain cycled PT‐COFs. a) PXRD patterns. b) N_2_ adsorption/desorption curves. c) Ionic conductivity of PT‐COFs‐QSSEs. d–i) SEM surface morphology images of PTCDA anode and CuPBA cathode. j–o) SEM cross‐sectional morphology images of PTCDA anode and CuPBA cathode. Insets are zoomed‐in images. p,q) XPS spectra of Ca *2p* and O *1s* of anode and cathode surfaces.

To better understand the status of the electrolyte, anode, and cathode of the cells after cycling, we conducted post‐mortem studies. First, we conducted ex situ FTIR of PT‐COFs‐QSSEs, anode, and cathode (Figure S18, Supporting Information). We found that there were no noticeable changes, such as shift or generation of shoulder peaks, on the peaks for –NH_2_, C═O, and C–N from the PT‐COFs‐QSSEs and C═O, C═C, and C–O from the anode and C≡N from cathode after 300 cycles at 0.1 A g^−1^ and 1000 cycles at 1 A g^−1^, indicating the high stability of PT‐COFs‐QSSEs and electrodes. First, we focused on thoroughly studying the stability of the PT‐COFs‐QSSEs. We conducted the following experiments: PXRD, N_2_ adsorption/desorption, and ionic conductivity measurement of PT‐COFs‐QSSEs after battery testing at 0.1 A g^−1^ for 300 cycles, and compared the results with those from pristine samples. The PXRD pattern of cycled PT‐COFs showed clear peaks that matched the pristine sample (**Figure** **5**a). Meanwhile, the cycled PT‐COFs kept a pore diameter of 1.3 nm and BET surface area of 158 m^2^ g^−1^, comparable to those of pristine PT‐COFs (1.3 nm and 192 m^2^ g^−1^; Figure 5b). Furthermore, we prepared new PT‐COFs‐QSSEs using the cycled PT‐COFs and measured ionic conductivity to see nearly the same Nyquist plot and ionic conductivity of 0.40 mS cm^−1^ at r.t. (Figure 5c). All these studies show the high stability of PT‐COFs as electrolytes for the fabrication of high‐performance QSS CIBs.

Although the stability of PTCDA anode and CuPBA cathode was known,^[^ ^17^, ^52^ ^]^ we checked on them to see if any dissolution had happened, or a passivation layer had been formed. First, we disassembled cells and characterized the chemical stabilities of the electrodes. As studied by the previous works, they were quite stable, showing nearly the same FTIR peaks. These corresponding characterization peaks kept at the same wavenumber after 300 cycles at 0.1 A g^−1^ and 1000 cycles at 1 A g^−1^. Then, we characterized the surface and cross‐sectional morphologies of pristine and post‐cycling electrodes using SEM. After cycling, both anode and cathode surface morphologies exhibited an aggregation phenomenon, indicating the existence of an interphase layer after cycling, presumably due to the reaction between calcium salt and PC during the initial electrochemical activation process (Figure 5d–i; Figures S19 and S20, Supporting Information). Notably, the dissolution issues of both electrodes did not happen after cycling. The SEM observations showed that both electrodes’ surfaces look clean and nice. However, the XPS analysis on both electrodes’ surfaces showed the appearance of calcium peaks after cycling (Figure 5p,q; Figure S21 and S22, Supporting Information). Two strong Ca peaks at 351.2  and 347.6 eV appeared in both the anode and cathode after cycling, corresponding to Ca‐2*p*_1/2_ and Ca‐2*p*_3/2_ (Figure 5p,q). Meanwhile, both the XPS spectra of O*1s* showed an increased intensity of peaks at 531.7 eV corresponding to the C–O^–^ group, indicating the increased content of C–O^–^ groups in the interphases. Considering the evolution of calcium and oxygen peaks shown in both electrode surfaces after cycling, the main component of both interphases was deduced as CaCO_3_.

Cross‐sectional SEM observations showed pretty much the same morphologies after cycling tests, indicating the insertion/de‐insertion of Ca^2+^ during the cycling process had a negligible effect on electrodes’ structures (Figure 5j–o; Figures S23 and S25, Supporting Information). Energy‐dispersive spectrometry (EDS) mapping was employed to proceed with the element analysis of the anode and cathode after different charge/discharge cycles (Figures S24 and S26, Supporting Information). Compared to the pristine electrodes, the calcium element mapping signal appeared in both the anode and cathode cross section after cycling, indicating the existence of calcium insertion/desertion during the charge/discharge process. In particular, calcium atomic percentage in both electrodes exhibited low content after different cycles at 0.1 and 1 A g^−1^, which was attributed to the initial electrochemical activation process. Furthermore, the relatively low calcium content, < 1% in the anode and < 1.9% in the cathode after cycling, and the almost unchanged content ratio of other elements indicated the anode and cathode structure integrity during the operation period (Tables S8 and S9, Supporting Information). The stable electrode structure supports the long‐term operation of a full calcium ion cell with PT‐COFs‐QSSEs.

## 3 Conclusion

In conclusion, we prepared quasi‐solid‐state electrolytes with two types of redox PT‐COFs and PQ‐COFs. The PT‐COFs‐QSSEs with richer and higher‐density carbonyl groups exhibited calcium ion conductivity of 0.46 mS cm^−1^ at r.t. The carbonyl groups were deduced as electrochemical active sites for fast calcium ion conduction by ex situ FTIR spectra and MEP simulation. The full calcium ion cell with PT‐COFs‐QSSEs exhibited a capacity of 155 mAh g^−1^ at the current density of 0.15 A g^−1^ (1 C) and kept a capacity retention of 74.6% over 1000 cycles at 1 A g^−1^, which is the best performance in a reported solid‐state calcium ion battery. This work is the first attempt to construct a COF‐based quasi‐solid‐state electrolyte for calcium ion batteries. The redox‐active carbonyl groups in the COF skeleton are demonstrated as efficient cation‐bonding sites for transportation, and the prepared quasi‐solid‐state electrolyte is beneficial for keeping the electrode structure stable, which sets up a new strategy for the construction of solid‐state electrolytes in rechargeable batteries.

This attempt demonstrates the feasibility of redox COF electrolytes in quasi‐solid‐state CIB, which is a good reference for further construction and optimization of all‐solid‐state rechargeable batteries.

## Conflict of Interest

The authors declare no conflict of interest.

## Supporting information



Supporting Information

Supplemental Video 1

## Data Availability

The data that support the findings of this study are available from the corresponding author upon reasonable request.
