# Supplementary material for: High‐Performance Quasi‐Solid‐State Calcium‐Ion Batteries from Redox‐Active Covalent Organic Framework Electrolytes
Source: Adv Sci (Weinh). 2025 Nov 16;13(7):e12328. doi: 10.1002/advs.202512328 (PMC12866863; doi:10.1002/advs.202512328)
Supplement: Supplementary file 1 — Supporting Information [file ADVS-13-e12328-s002.docx]

**Supporting Information**

**High-Performance Quasi-Solid-State Calcium-Ion Batteries from Redox-Active Covalent Organic Framework Electrolytes**

Zhuoyu Yin^1^, Jixin Wu^2^, Ye Tian^1^, Yufei Yuan^1^, Muhua Gu^1^, Lei Cheng^2^, Yanming Wang^3^, Yoonseob Kim^1,4,^*

^1^Department of Chemical and Biological Engineering, The Hong Kong University of Science and Technology, Clear Water Bay, Kowloon, Hong Kong SAR, China

^2^University of Michigan - Shanghai Jiao Tong University Joint Institute, Shanghai Jiao Tong University, Shanghai 200240, People’s Republic of China

^3^Global Institute of Future Technology, Shanghai Jiao Tong University, Shanghai 200240, People’s Republic of China

^4^Energy Institute, The Hong Kong University of Science and Technology, Hong Kong SAR, China

*To whom correspondence should be addressed: yoonseobkim@ust.hk

**Table of Contents**

1. Chemicals and materials
2. Characterization instruments
3. Synthesis procedures
4. Preparation of quasi-solid-state electrolytes (QSSEs)
5. Ionic conductivity, linear sweep voltammetry and transference number measurement
6. Electrochemical properties testing
7. Simulation information
8. **Chemicals and materials**

9,10-Phenanthrenequinone (PQ), 2,4,6-triformylphloroglucinol (TFG), pyrene, mesitylene, ruthenium (III) chloride hydrate, dichlorodicyanobenzoquinone, sodium periodate, propylene carbonate (PC), perylenetetracarboxylic dianhydride (PTCDA) and sodium dithionite are purchased from Energy Chemical. Sodium carbonate, sodium sulfide nonahydrate, sodium hydroxide, ammonium chloride, and 1,4-dioxane are purchased from Shanghai Aladdin Biochemical Technology Co., Ltd. Cupric nitrate trihydrate and potassium ferricyanide are supplied by 3A Materials^®^. Sulfuric acid, nitric acid, and acetic acid are purchased from Sigma-Aldrich. All reagents are used without any purification.

1. **Characterization instruments**

^1^H NMR spectra were measured on a Bruker AVII 400 MHz NMR Spectrometer. The FTIR spectra were measured by the Vertex 70 Hyperion 1000 (Bruker). Powder x-ray diffraction (PXRD) data were tested on Analytical x-ray diffractometer (Cu Kα radiation λ = 1.54056 Å). Scanning Electron Microscopy (SEM) was taken by using JSM-6700F (JEOL) equipped with Energy dispersive X-ray spectroscopy (EDX, 50kV). Transmission Electron Microscopy (TEM) was taken by using JEOL 2010F. The nitrogen adsorption-desorption isotherms were recorded on BELSORP MINI X analyzer at 77 K. The thermogravimetric analysis (TGA) was tested ranging from 25 °C to 800 °C with a heating rate of 10 °C min^–1^. The X-ray photoelectron spectroscopy (XPS) is measured by Kratos Axis Supra^+^ multi-technique surface analysis system.

1. **Synthesis procedures**


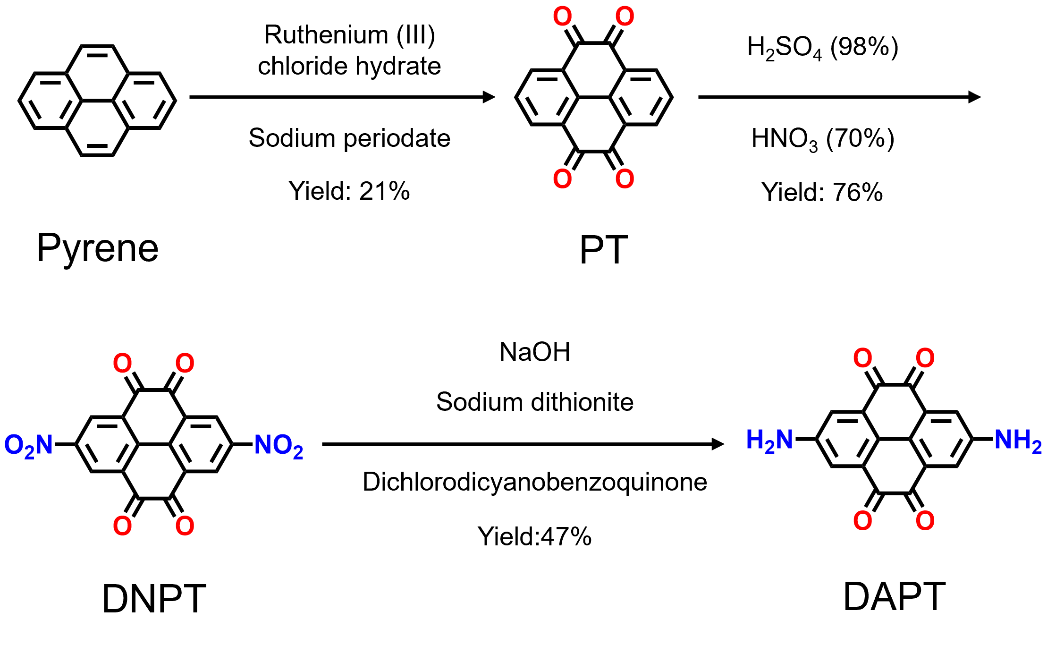


**Scheme S1. Synthetic schemes for pyrene-4,5,9,10-tetraone (PT), 2,7-dinitropyrene-4,5,9,10-tetraone (DNPT), and 2,7-diaminopyrene-4,5,9,10-tetraone (DAPT) starting from pyrene.**

- 1. **Synthesis of PT**

PT was synthesized according to the literature.^1^ Pyrene (4.0 g, 20 mmol) was added in a 500 mL round flask with a mixture of 80 mL dichloromethane (DCM) and 80 mL acetonitrile. Then, sodium periodate (35.0 g, 164 mmol), deionized water (100 mL), and ruthenium (III) chloride hydrate (0.5 g, 0.42 mmol) were added to the mixture. The reaction mixture was kept at 37 °C with stirring overnight. After the reaction, the upper organic solution was collected and the remaining suspension was extracted by DCM 3 times. The extracted solution and collected upper solution were combined, and then the solvent was removed by rotary evaporation. The crude product was recrystallized from chloroform to get a yellow powder of PT (1.1 g) with a yield of 21 %. ^1^H NMR (400 MHz, DMSO-d_6_, δ [ppm]), 8.33 (d, 4H), 7.75 (t, 2H).


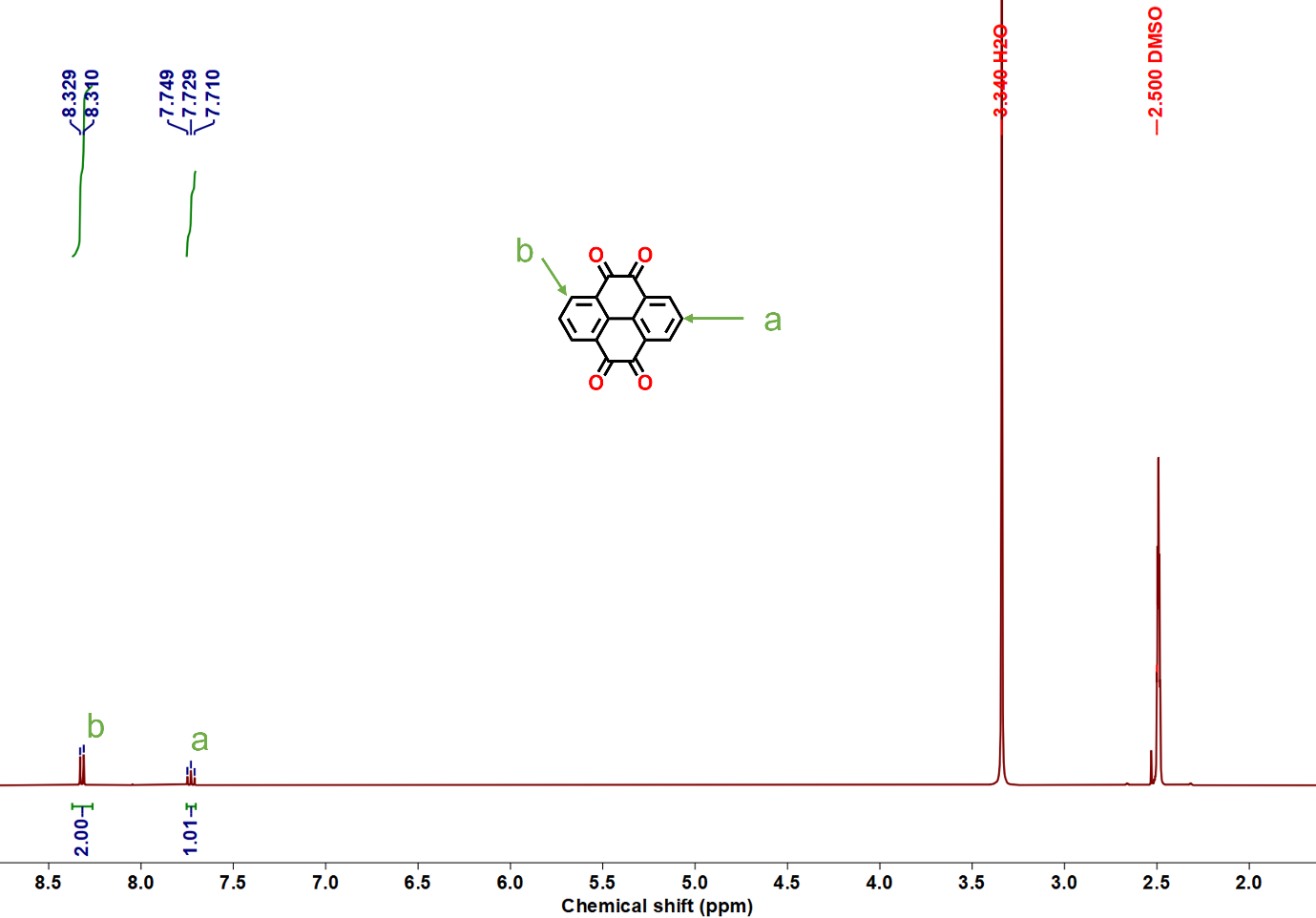


**Figure S1. 400 MHz ^1^H NMR spectrum of PT in DMSO-d_6_.**

- 1. **Synthesis of DNPT**

DNPT was synthesized according to the literature.^2-3^ PT (0.63 g, 2.4 mmol) was added in a 100 mL round flask. Then, concentrated HNO_3_ (70%, 8 mL) and concentrated H_2_SO_4_ (98%, 32 mL) were added into the flask. After keeping the mixed solution at 90 °C for 4 h, the reaction solution was poured into deionized water (200 mL). A saturated NaHCO_3_ solution was used to neutralize the mixing solution to pH ~ 7. After filtration, the filtrate was dried under vacuum at 65 °C for 24 h to get DNPT (0.64 g), with a yield of 76%. ^1^H NMR (400 MHz, DMSO-d_6_, δ [ppm]), 8.88 (s, 4H).


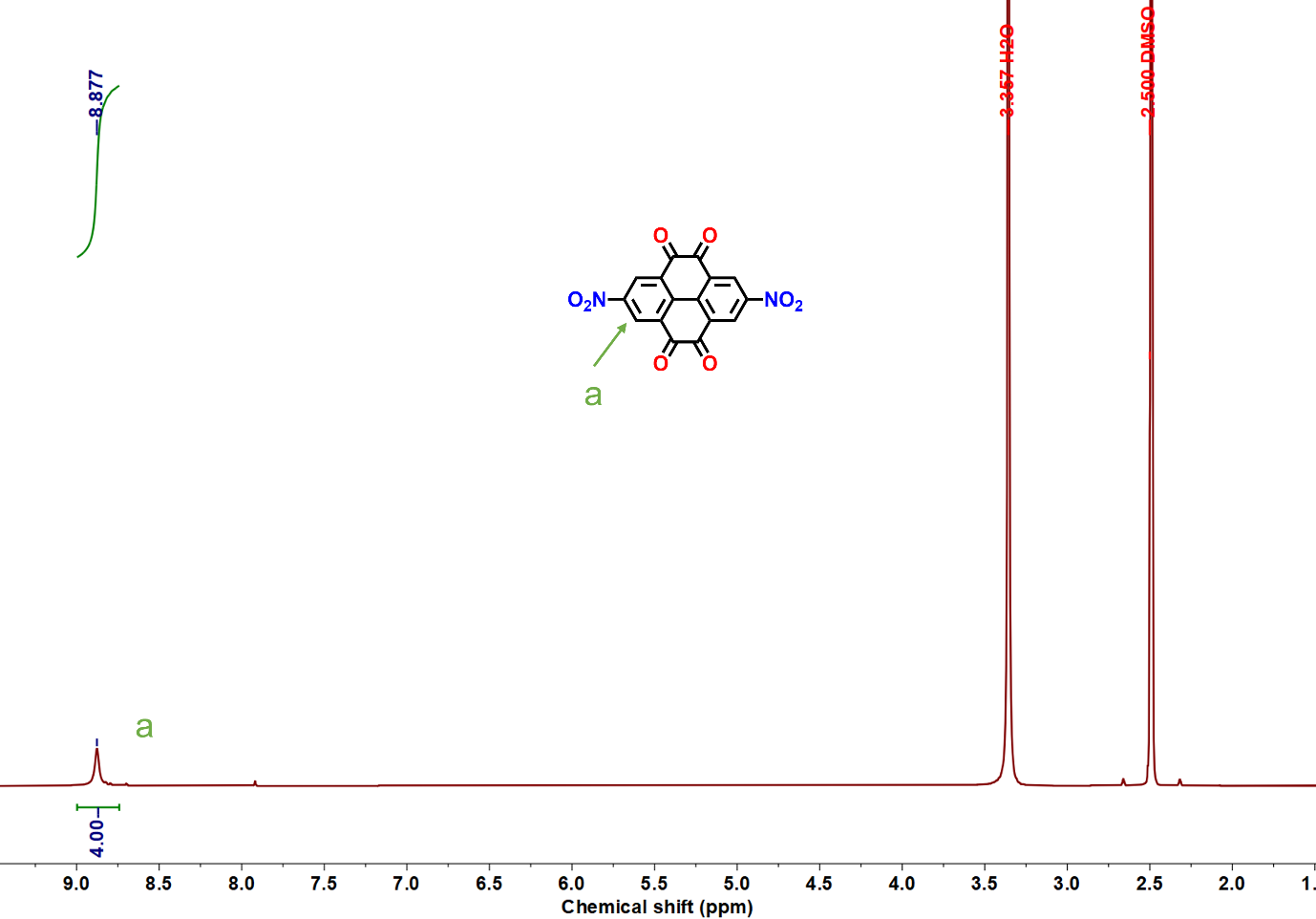


**Figure S2. 400 MHz ^1^H NMR spectrum of DNPT in DMSO-d_6_.**

- 1. **Synthesis of DAPT**

DAPT was synthesized according to literature ^2-3^. DNPT (0.64 g, 1.8 mmol), NaOH (5.7 g), Na_2_S_2_O_4_ (2.9g) and deionized water (100 mL) were added in a 200 mL round flask. The mixture solution was kept at 50 °C for 15 mins. Then, 300 mL of saturated NH_4_Cl solution was added to the reaction solution. After filtration, the black crude product was dried at room temperature (r.t.) in a vacuum overnight. The crude product (0.38 g), dichlorodicyanobenzoquinone (1.25 g) and methanol (30 mL) were added to a 100 mL flask and the solution was kept at 35 °C overnight. After washing with ethyl acetate and filtration, the black power was dried at 60 °C to afford DAPT (0.32 g), with a yield of 47%. ^1^H NMR (400 MHz, DMSO-d_6_, δ [ppm]): 7.35 (s, 4H), 5.96 (s, 4H).


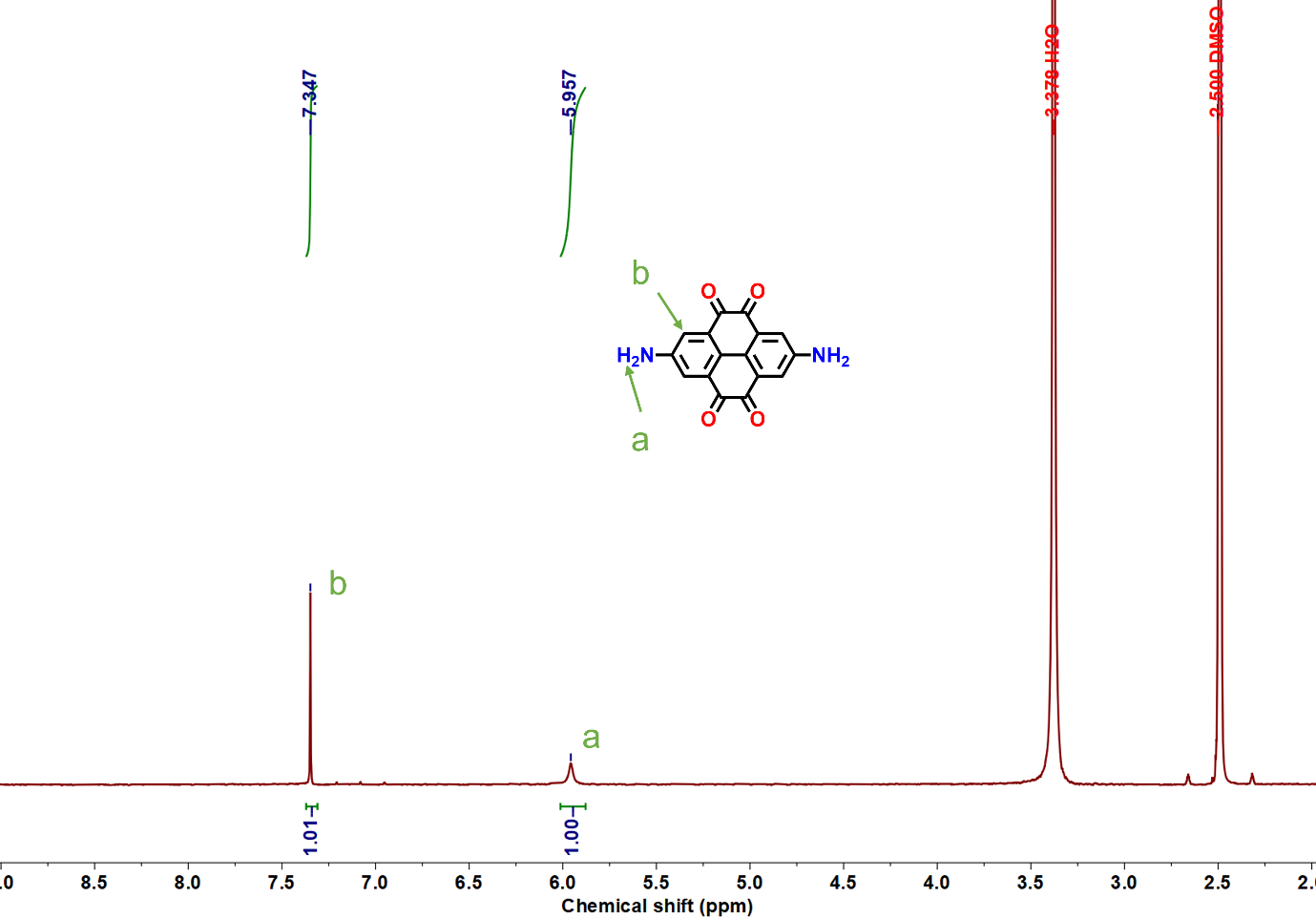


**Figure S3. 400 MHz ^1^H NMR spectrum of DAPT in DMSO-d_6_**.

- 1. **Synthesis of PT-COFs**

TFG (10.5 mg, 0.05 mmol), DAPT (21.9 mg, 0.075 mmol), mesitylene (0.9 mL), 1,4-dioxane (0.3 mL), and aqueous acetic acid (0.2 mL, 6 M) were added in a 15 mL Pyrex tube. After ultrasonication for 30 mins and three freeze-pump-thaw, the tube was kept at 120 °C for 3 days. The black powder was washed with DMF and acetone to get PT-COFs (27 mg), with a yield of 91%.


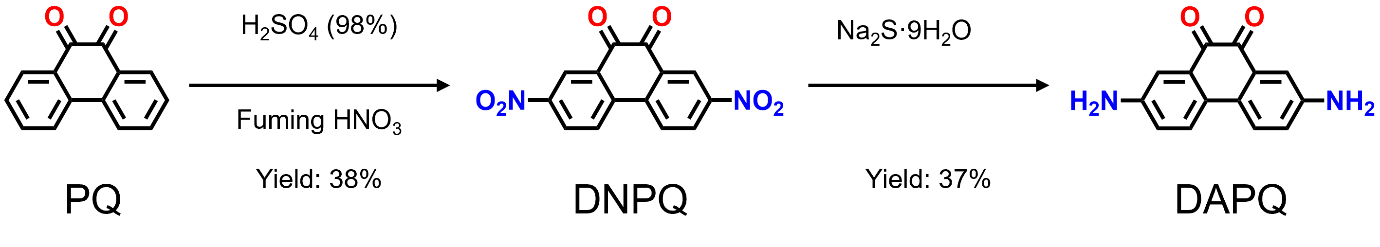


**Scheme S2. Synthetic schemes for 2,7-dinitro-9,10-phenanthrenequinone (DNPQ) and 2,7-diamino-9,10-phenanthrenequinone (DAPQ) starting from 9,10-phenanthrenequinone (PQ).**

- 1. **Synthesis of 2,7-Dinitro-9,10-phenanthrenequinone (DNPQ)**

DNPQ was synthesized according to the reported procedure.^4^ First, PQ (2.0 g, 9.6 mmol) was added in a 100 mL round-bottomed flask. Then, fuming HNO_3_ (28 mL) and concentrated H_2_SO_4_ (98%, 2.8 mL) was slowly added dropwise into the flask and the reaction solution was kept at 90 ℃ with stirring for 3 h. After that, the resulting solution was poured into 500 mL of deionized water. The statured Na_2_CO_3_ solution was employed to adjust the pH of the mixture solution to neutral. After filtration, the crude product was recrystallized from acetic acid dry at r.t. and vacuumed overnight to obtain yellow powder of DNPQ (1.15g), with a yield of 38%. ^1^H NMR (400 MHz, CDCl_3_-d_3_, δ [ppm]): 8.36 (d, 2H), 8.66 (dd, 2H), 9.09 (d, 2H).


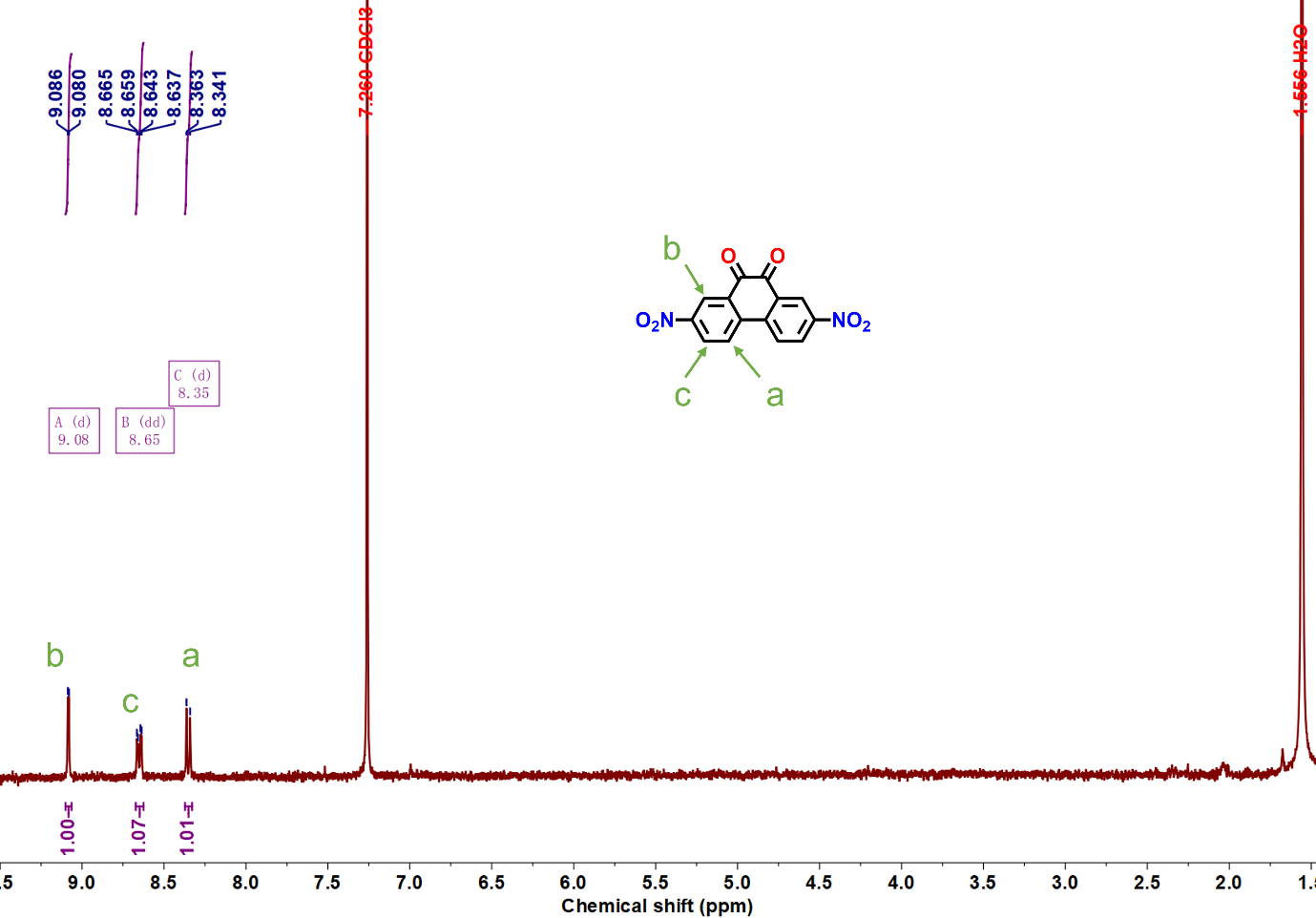


**Figure S4. 400 MHz ^1^H NMR spectrum of DNPQ in CDCl_3._**

- 1. **Synthesis of 2,7-diamino-9,10-phenanthrenequinone (DAPQ)**

DAPQ was synthesized according to a literature procedure.^5^ DNPQ (1 g, 3.35 mmol) and methanol (20 mL) were added in a 100 mL two-necked flask with stirring. Then, Na_2_S·9H_2_O (0.7 g) and Na_2_CO_3_ (0.72g) were added to the mentioned solution. The mixed solution was heated at 90 ℃ with an N_2_ atmosphere overnight. When the reaction finished, the reaction solution was diluted with 50 mL of deionized water and filtrated to get the crude product. The crude product was purified by recrystallization from methanol to get black power of DAPQ (0.37 g), with a yield of 37%. ^1^H NMR (400 MHz, DMSO-d_6_, δ [ppm]): 7.63 (d, 2H), 7.07 (d, 2H), 6.84 (dd, 2H), 5.53 (s, 4H).


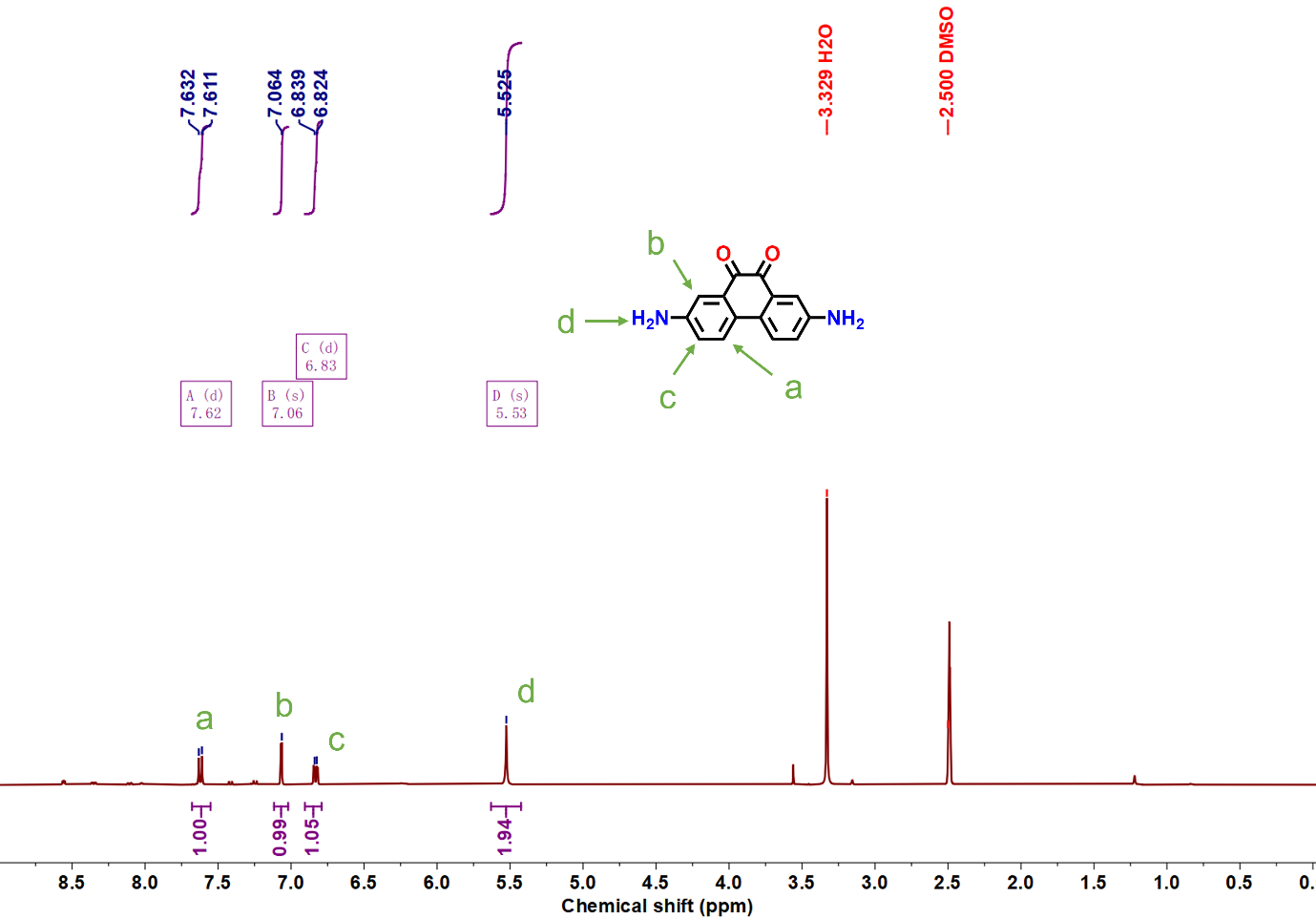


**Figure S5. 400 MHz ^1^H NMR spectrum of DAPQ in DMSO-d_6_.**

- 1. **Synthesis of PQ-COFs**

TFG (10.5 mg, 0.05 mmol), DAPQ (17.9 mg, 0.075 mmol), mesitylene (0.9 mL), 1,4-dioxane (0.3 mL), and aqueous acetic acid (0.2 mL, 6 M) were added in a 15 mL Pyrex tube. After ultrasonication for 30 minutes and three freeze-pump-thaw cycles, the glass tube was sealed and heated to 120 ℃ for 3 days. The black precipitate was washed by DMF and acetone overnight to obtain the PQ-COFs (22 mg) with a yield of 89%.

1. **Preparation of quasi-solid-state electrolytes (QSSEs)**

**PT-COFs-QSSEs:** PT-COFs (100 mg) and calcium (II) bis(trifluoromethanesulfonimide) (50 mg) were dispersed/dissolved in 5 mL methanol, treated with ultrasonication for 10 mins. The methanol was evaporated and the mixed powder was dried at 120 ℃ for 1 h. After that, the mixed powder (60 mg) was placed in a stainless-steel die (15 mm diameter) and then pressed at ca. 20 MPa for 5 mins to prepare the pellet, and the thickness of the prepared pellet is 30 μm. Before assembling the coin cell, propylene carbonate (PC; 10 μL, 20 wt.%) was added to the prepared pellet.

The PQ-COFs-QSSEs were prepared following the same procedures.

1. **Ionic conductivity, linear sweep voltammetry and transference number measurement**

The impedance of the prepared QSSEs were evaluated by Electrochemical Impedance Spectroscopy (EIS) with a frequency range from 1 MHz to 1 Hz (Autolab PGSTAT204). Before the testing, the thickness of the pellet was recorded. After adding PC, the prepared PT-COFs-QSSEs was assembled in a coin cell (2032) with stainless steel as both electrodes. Ionic conductivity (*σ*, mS cm^–1^) of prepared QSSEs can be calculated by the following equation:

$$\sigma=\frac{L}{SR}$$

where *L* is the thickness of the electrolyte pellet, *S* is the surface area of the pellet, and *R* is the impedance tested by EIS.

The activation energy was calculated by Arrhenius plot using the below equation:

$$\sigma=Ae^{\frac{{-E}_{a}}{RT}}$$

where *σ* is the ionic conductivity of QSSEs at different temperatures, *A* is the pre-exponential factor, *E_a_* is the activation energy for ion transport, *R* is the gas constant, and *T (K)* is the corresponding temperature.

The linear sweep voltammetry (LSV) of prepared QSSEs was conducted through a three-electrode configuration with Pt as both working and counter electrode, and Ag/Ag^+^ as reference electrode in a Swagelok cell at a scan rate of 0.2 mV s^–1^. Pt is widely recognized as an electrochemically inert electrode for a wide potential window and minimizes confounding Faradaic processes from the electrode itself, ensuring that the measured current is primarily indicative of electrolyte decomposition rather than electrode corrosion or passivation. Meanwhile, Ag/Ag^+^ reference electrode was employed in this organic system to define the potential of Pt working electrode accurately. The commercial Ag/Ag^+^ reference electrode had a filling solution of 10 mM AgNO_3_ in acetonitrile with 100 mM tetrabutylammonium hexafluorophosphate as supporting electrolyte salt.

The Ca^2+^ transference number ($t_{{Ca}^{2+}}$) of prepared QSSEs was evaluated by Bruce−Vincent−Evans technique in a Ca|QSSEs|Ca cell at room temperature.^6^ $t_{{Ca}^{2+}}$ was calculated following the formula:

$$t_{{Ca}^{2+}=\frac{i_{ss}(\Delta V-i_{0}R_{0})}{i_{0}(\Delta V-i_{ss}R_{ss})}}$$

where *ΔV* (30 mV) is applied DC voltage, *i*_0_ and *i*_ss_ are the initial state and steady state current, *R*_0_ and *R*_ss_ are the initial state and steady state impedance tested by EIS from 1 MHz to 0.1 Hz, respectively.

1. **Electrochemical properties testing**
   1. **Synthesis of Cu-coordianted Prussian blue analogue (CuPBA)**

The CuPBA was synthesized according to the literature.^7^ Cu(NO_3_)_2_·3H_2_O (2.56 g, 10 mmol) and K_3_Fe(CN)_6_ (1.64 g, 5 mmol) were dissolved in deionized water (10 mL) respectively. After ultrasonication for 15 mins, both solutions were added dropwise to 60 mL of deionized water, and this process lasted around 60 mins. The resulting solution was kept overnight. After filtration, the filtrate was dried at r.t. in a vacuum for 2 days to get the CuPBA powder.

- 1. **Preparation of PTCDA|QSSEs|CuPBA full calcium ion battery**

Perylenetetracarboxylic dianhydride (PTCDA) and CuPBA were selected as anode and cathode active materials respectively. The anode/cathode active materials (70 wt.%), super P (20 wt.%), PVDF (10 wt.%) and NMP were mixed to prepare a slurry and then coated on Al foil. The coated electrodes were dried at 70 ℃ overnight, and the loading of active materials is 1.0 and 1.5 mg cm^–2^ for the cathode and anode, respectively. The thickness of prepared electrode is around 35 μm. The PTCDA anode, COFs-QSSEs and CuPBA cathode were assembled in a coin cell (2032) and kept unmoved for 12 h. The theoretical capacity of CuPBA cathode is 170 mAh g^–1^.^8^ The battery performance was evaluated by the Neware battery test system at r.t. with a voltage range of 0–1.8 V. The batteries’ specific capacity (SC, mAh g^–1^) was calculated by following formula:

$$SC=\frac{Q}{m}$$

where *Q (mAh)* was the charge/discharge capacity tested by Neware battery test system and *m (g)* was the loading of cathode active materials.

The volumetric energy density (Wh L^–1^) and gravimetric energy density (Wh kg^–1^) are calculated by following formula:

$$volumetric energy density=\frac{V_{A}\times Q}{{Vol}_{coin cell}}$$

$$gravimetric energy density=\frac{V_{A}\times Q}{m_{coin cell}}$$

Where *V_A_* is the average discharge voltage, *Vol_coin cell_* and *m*_coin cell_ are the volume (*L*) and mass (*kg*) of the assembled full cell. The net mass of a coin cell (2032) is 5.08 g, including one positive electrode shell, one negative electrode shell, one spring, and two stainless steel gaskets. The mass of one assembled full cell was 5.19 g.

1. **Calculation information**

Composite electrolyte models of PT- and PQ-COFs-QSSEs were constructed to understand the Ca^2+^ transport behaviors. First, the smallest periodic unit cells of pure COFs were modeled in MedeA, followed by building 4×4×8 supercells. Using the LAMMPS simulation package, 2 ns NVT and 3 ns NPT relaxations were performed at 473 K to obtain the relaxed PTCOF and PQCOF structures. Composite models were created by randomly placing Ca²⁺, TFSI⁻, and PC molecules into the COF pores, with molecular number ratios of PQCOF_unit cell_ : Ca(TFSI)₂ : PC = 1 : 0.85 : 3 and PTCOF_unit cell_  : Ca(TFSI)₂ : PC = 1 : 1 : 3*.* Subsequently, the entire system underwent 1 ns NVT and 4 ns NPT relaxations at 473 K, resulting in fully optimized configurations.

Interaction parameters were generated using the Polymer Consistent Force Field (PCFF+) in MedeA, accurately describing bonded (bond, angle, dihedral, improper) and non-bonded (lj-class2 and coulombic) interactions. Except for the point charges of electrolyte anions and cations, all other matching parameters were directly adopted. The original point charges of electrolyte ions were scaled by 0.7, with the final charge details listed in Table S2.

After optimization, the composite configurations of PT-/PQCOFs were subjected to 50 ns NVT dynamics at 473 K. RDF analysis was perfomed by LAMMPS built-in calculation algorithm by setting bins to 450 and cutoff larger than force cutoff range on both systems. In our study, it is observed that *g(r)* converges to 1 when r exceeds 10 Å. Thus, we set the cutoff at a larger value of 15 Å to ensure the accuracy of calculations across all simulation scenarios. Each dataset is derived from three independent parallel simulations, with time averaging performed over the last 30 ns of trajectories.

The DFT calculations of HOMO–LUMO (HOMO: highest occupied molecular orbital; LUMO: lowest unoccupied molecular orbital) values of monomers were carried out by using Gaussian 16 package and B3LYP molecular geometry.^9^ The visualization of HOMO–LUMO of DAPT and DAPQ monomers were generated with GaussView6.^10^ Simulated XRD pattern was generated from the unit cell in the Reflex module.

**Table S1.** **Comparison of ionic conductivities in QSSE samples having different ratios of COF and calcium salts and different amounts of solvent.**

| **Entry** | **PT-COFs** | **Ca salt** | **Solvent** | **Ionic conductivity**  **(mS cm^–1^) at r.t.** |
| --- | --- | --- | --- | --- |
| 1 | 50% | 50% | 0% | 1.4*10^–6^ |
| 2 | 25% | 75% | 0% | 3.4*10^–5^ |
| 3 | 41.7% | 41.7% | 16.6% PC | 6.8*10^–1^ |
| 4 | 38.5% | 38.5% | 23% PC | 1.1*10^0^ |
| 5 | 60.6% | 30.3% | 9.1% PC | 3.3*10^–2^ |
| **6^#^** | **55.6%** | **27.8%** | **16.6% PC** | **4.6*10^–1^** |
| 7 | 66.7% | 16.7% | 16.6% PC | 1.0*10^–2^ |
| 8 | 20.9% | 62.5% | 16.6% PC | 6.7*10^–2^ |
| 9 | 19.2% | 57.7% | 23.1% PC | 1.2*10^–1^ |
| 10 | 17.8% | 53.6% | 28.6% PC | 8.9*10^–1^ |
| 11 | 41.7% | 41.7% | 16.6% DME | 7.6*10^–2^ |
| 12 | 55.6% | 27.8% | 16.6% DME | 4.4*10^–3^ |
| 13 | 55.6% | 27.8% | 16.6% mixed solvent | 3.2*10^–2^ |
| 14 | 50% | 50% | 0% | 2.8*10^–7^ |
| 15 | 50% | 50% | 0% | 7.0*10^–8^ |

^#^Entry 6 is used as a main sample for the work. We optimized the amount of salt and PC in a way to minimize their addition.

Note: (1) Entry 1 to 13, the Ca salt is Ca (TFSI)_2_. Entry 14 and entry 15, the Ca salt is CaCl_2_ and Ca(NO_3_)_2_, respectively. For each entry, the total weight of PT-COFs and Ca salt is 60 mg and the ratio is weight percentage.

(2) DME, EC, DMC, and EMC stand for dimethoxyethane, ethylene carbonate, dimethyl carbonate, ethyl methyl carbonate, respectively. Mixed solvent had EC:PC:DMC:EMC=2:2:3:3 (v/v/v/v).


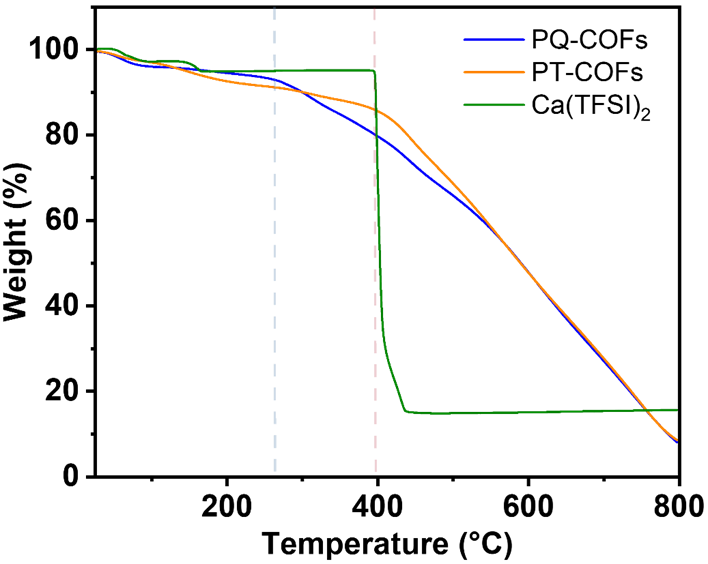


**Figure S6.** **Thermal stability of PQ-COFs, PT-COFs, and Ca(TFSI)_2_.**

**
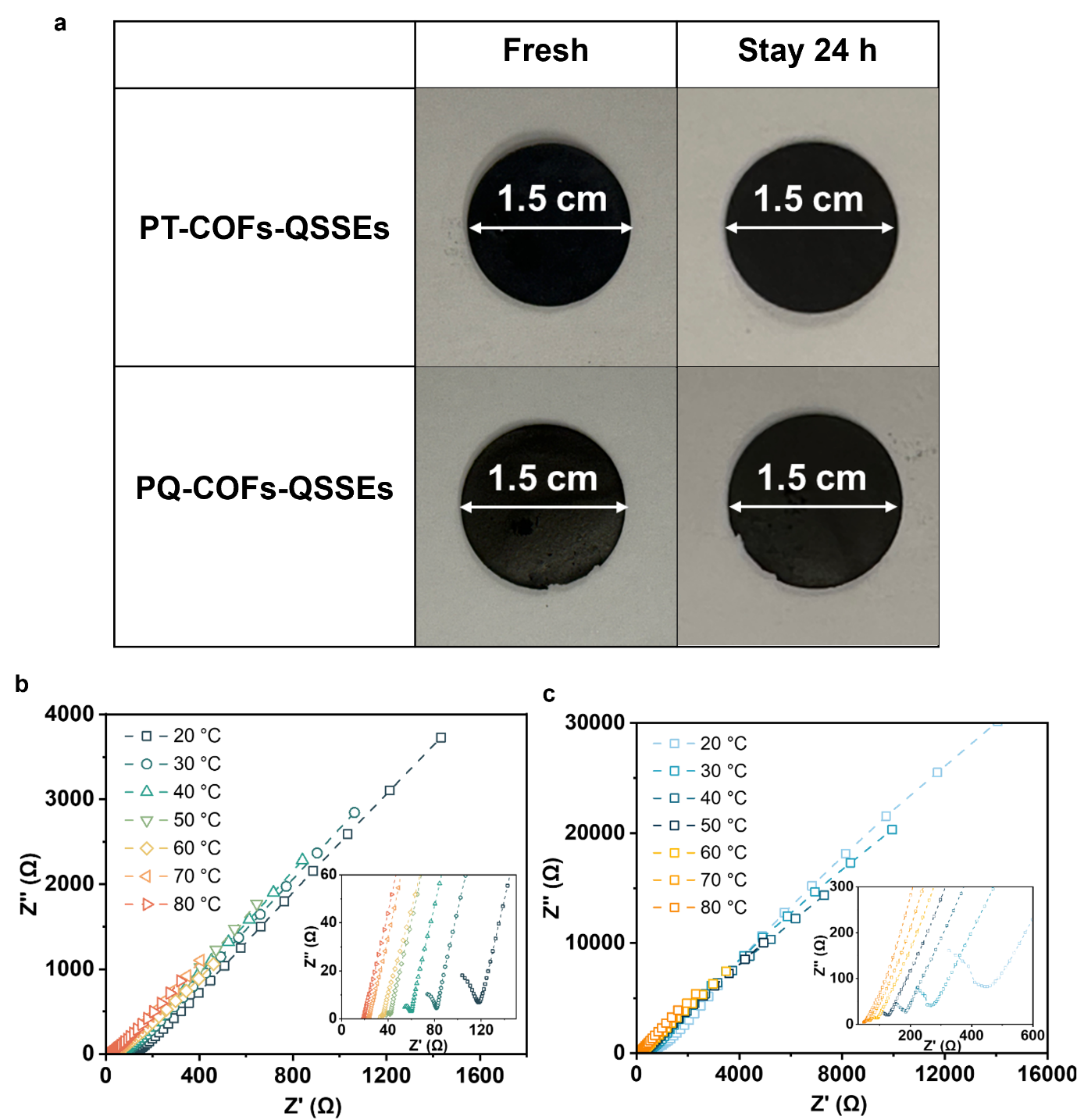
**

**Figure S7. a, Optical images of prepared PT-/PQ-COFs-QSSEs. Nyquist plots at different temperatures of (b) PT-COFs-QSSEs, (c) PQ-COFs-QSSEs in coin cell with stainless steel as both electrodes. The prepared QSSE pellets with a diameter of 1.5 cm and average thickness of 30 μm.**


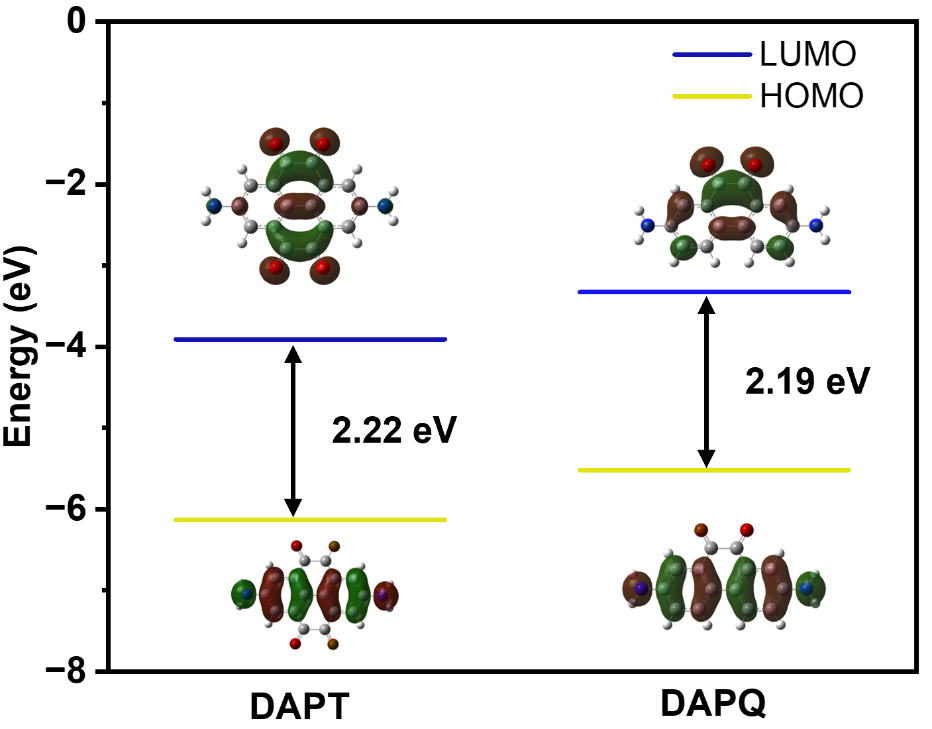


**Figure S8. HOMO–LUMO energy and orbitals profiles of DAPQ and DAPT monomers.**


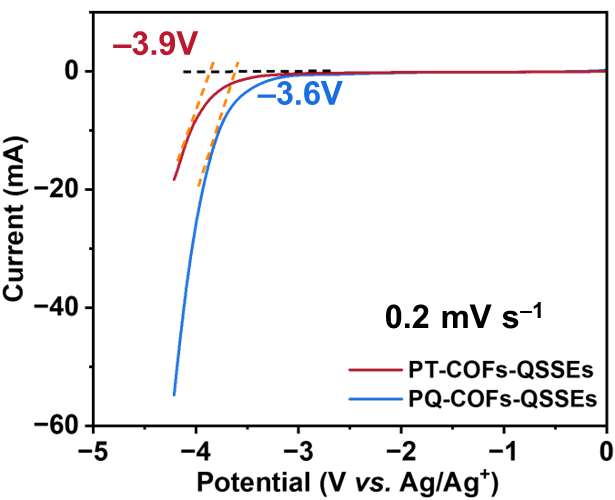


**Figure S9. Cathodic stability of PT-COFs-QSSEs and PQ-COFs-QSSEs at a scan rate of 0.2 mV s^–1^ in a three-electrode system with Pt as working and counter electrode, and Ag/Ag^+^ as reference electrode.**

**
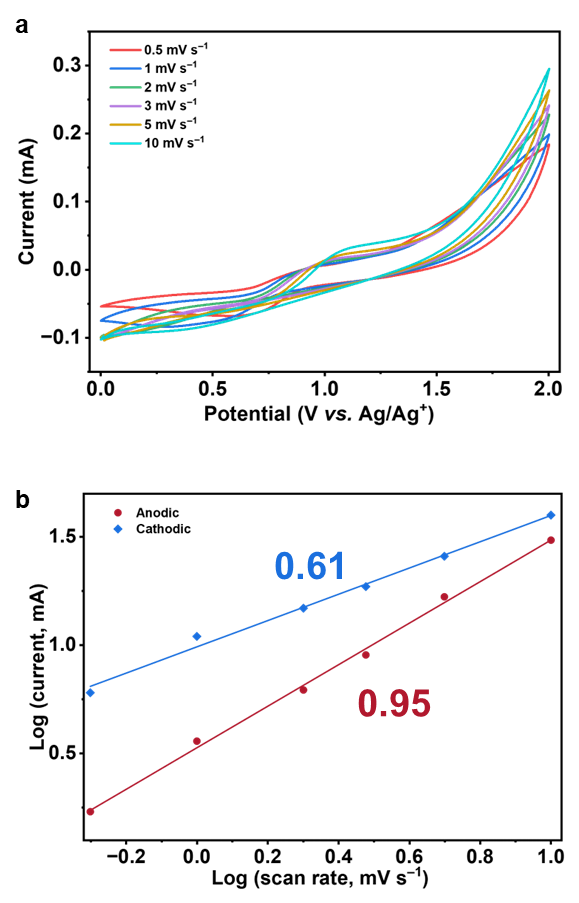
**

**Figure S10. a, CV curves of PT-COFs at various scan rates from 0.5 to 10 mV s^–1^. b, Relationship between log (*i*) vs. log (*v*) for the CV profiles of PT-COFs in Figure S10a.**

**
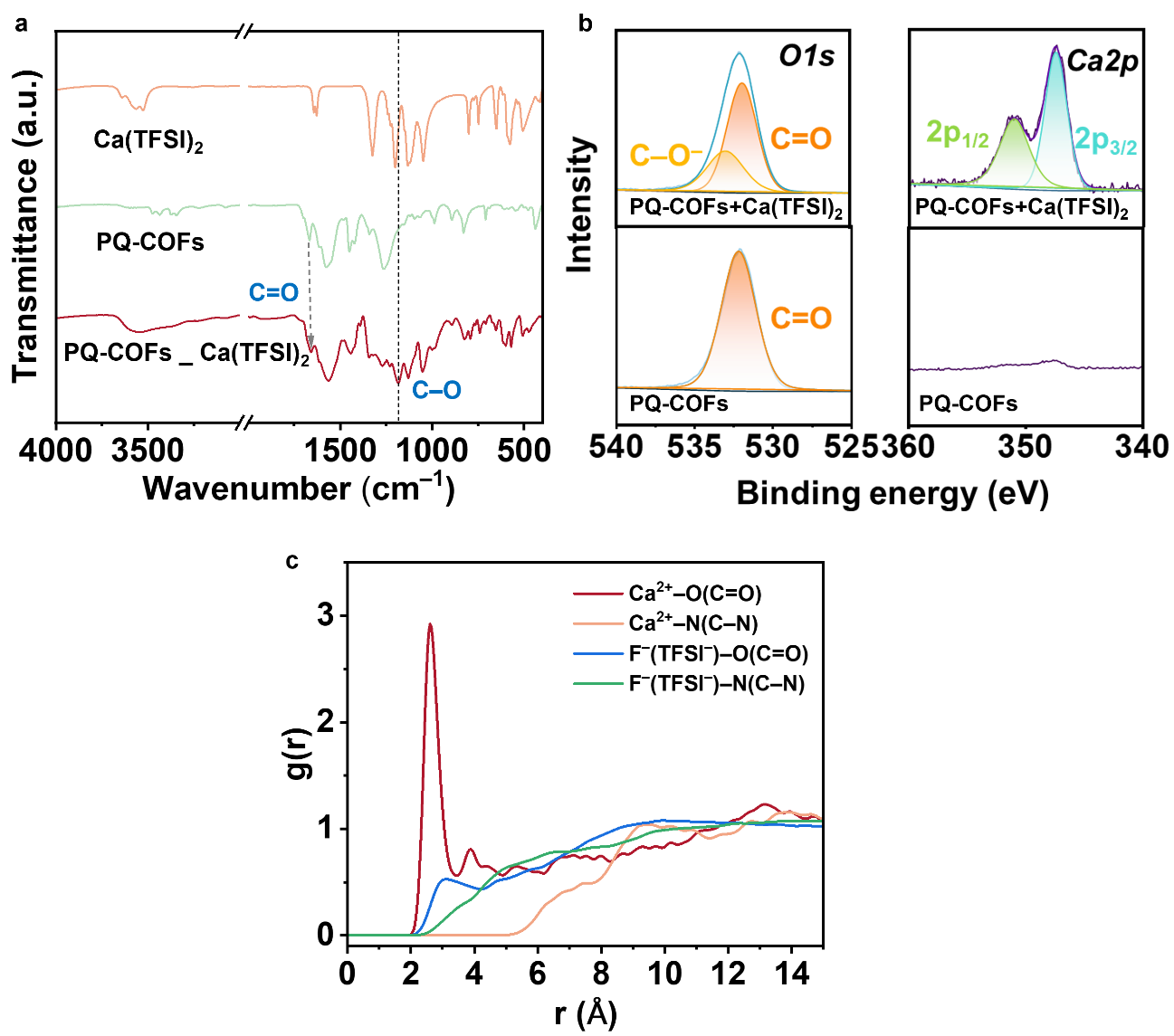
Figure S11. a, FTIR spectrum of PQ-COFs, Ca(TFSI)_2_ and their composite. b, XPS spectra of PQ-COFs and the composites. c, Radial distribution functions (RDF) of Ca^2+^ and TFSI^–^ ions in relation to redox centers (O, N atoms) in PQ-COFs.**

**Table S2. Static atomic point-charges of Ca(TFSI)_2_ used in PCFF+ model for MD simulation.**

| **Species** | **Charge(e)** |
| --- | --- |
| Ca | +1.400000 |
| C (TFSI**^–^**) | +0.420000 |
| F (TFSI**^–^**) | –0.112000 |
| S (TFSI**^–^**) | +0.448000 |
| O (TFSI**^–^**) | –0.294000 |
| N (TFSI**^–^**) | –0.588000 |
| H (TFSI**^–^**) | 0.252 |


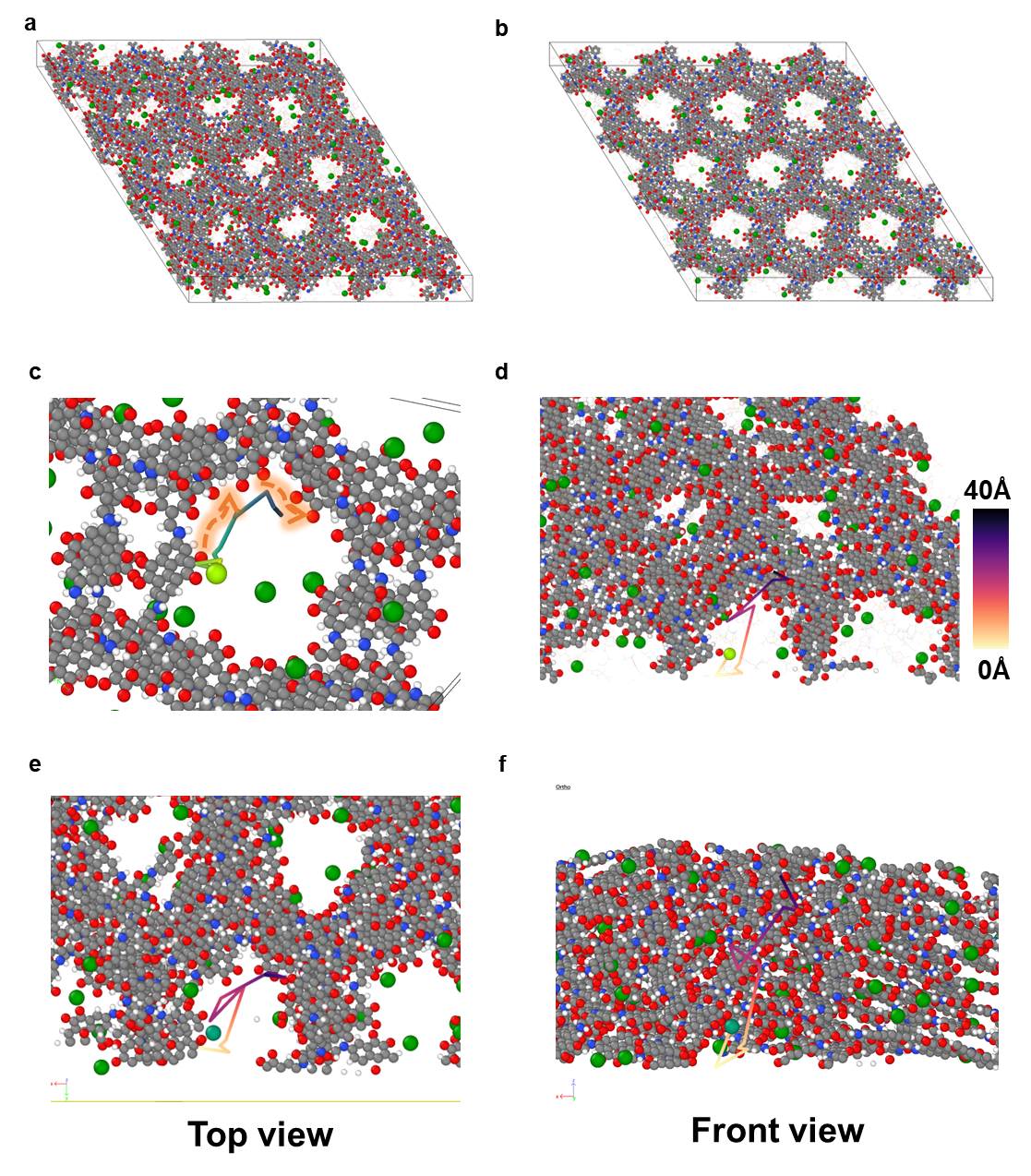


**Figure S12. Well relaxed configuration used in MD simulation (a) PT-COFs-QSSEs, (b) PQ-COFs-QSSEs. c, NVT calculations on the relaxed structure of the PT-COFs-QSSEs box to visualize the Ca^2+^ transport trajectories in the *x-y* direction from 8.5 to 54.5 ns. d, Illustrations of Ca^2+^ transport trajectories in the *z* direction from 8.5 to 54.5 ns in Figure S12c. Capture *z*-direction motion in Figure S12d from (e) top view; (f) front view. (Green: Ca^2+^, Slight green: labelled Ca^2+^, Black: C; Red: O; Blue: N; White: H).**


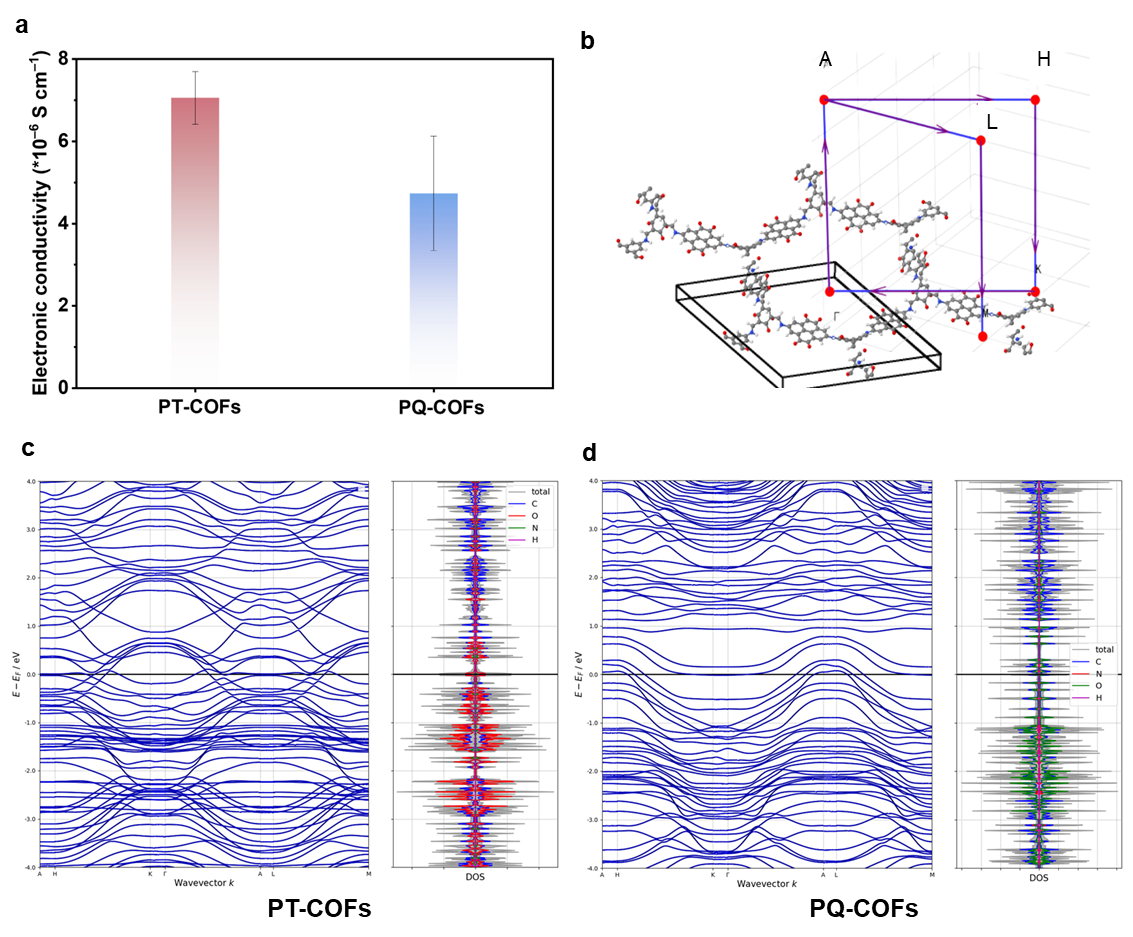


**Figure S13. a, Electronic conductivity of PT-COFs and PQ-COFs at room temperature. b, model of PT-COFs for DFT band structure calculations. DFT band structure of (c) PT-COFs, (d) PQ-COFs.**

**Table S3. DFT calculated band structure information of PT-COFs.**

| **PT-COFs** | | | |
| --- | --- | --- | --- |
| Spin Channel | <UP> | <DOWN> | <TOTAL> |
| Band Gap (eV) | 0.0037 | 0.0037 | 0.0037 |
| Eigenvalue of VBM (eV) | –2.5944 | –2.5944 | –2.5944 |
| Eigenvalue of CBM (eV) | –2.5907 | –2.5907 | –2.5907 |
| Fermi Energy (eV) | –2.5909 | –2.5909 | –2.5909 |

**Table S4. DFT calculated band structure information of PQ-COFs.**

| **PQ-COFs** | | | |
| --- | --- | --- | --- |
| Spin Channel | <UP> | <DOWN> | <TOTAL> |
| Band Gap (eV) | 0.0019 | 0.0019 | 0.0019 |
| Eigenvalue of VBM (eV) | –1.5946 | –1.5946 | –1.5946 |
| Eigenvalue of CBM (eV) | –1.5927 | –1.5927 | –1.5927 |
| Fermi Energy (eV) | –1.5930 | –1.5930 | –1.5930 |


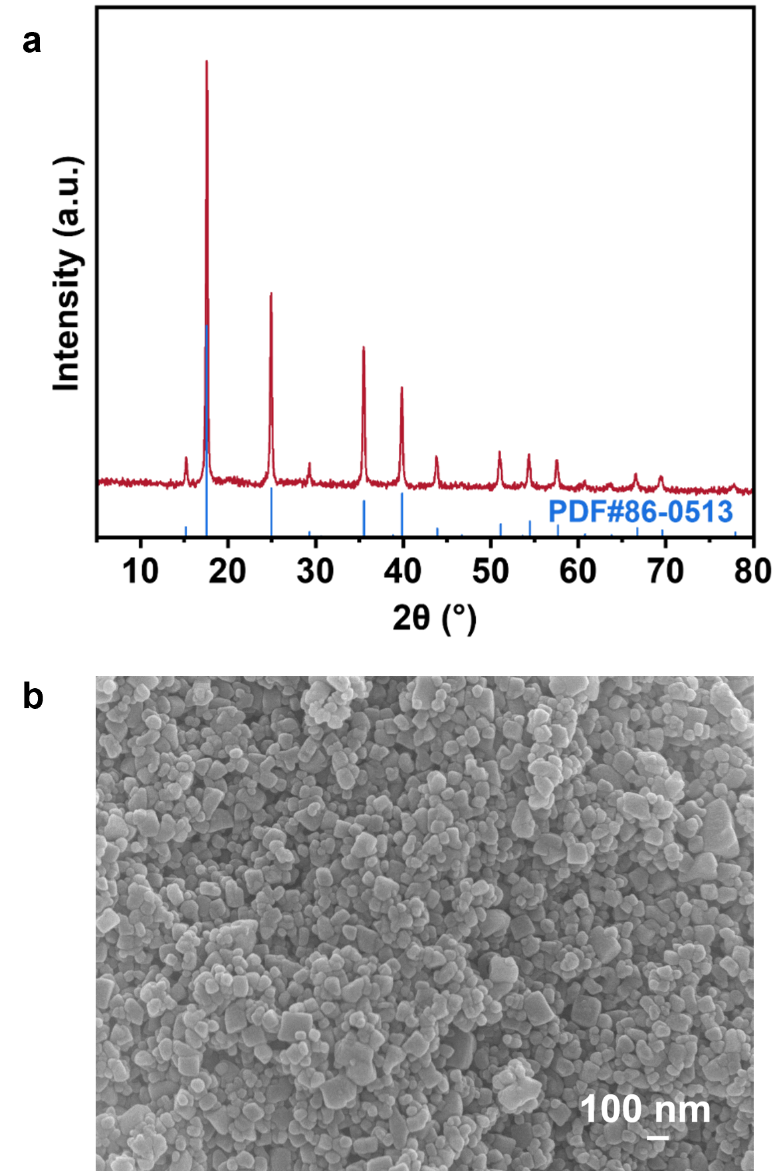


**Figure S14. CuPBA cathode characterization data. a, b**, **XRD pattern and SEM image, respectively.**


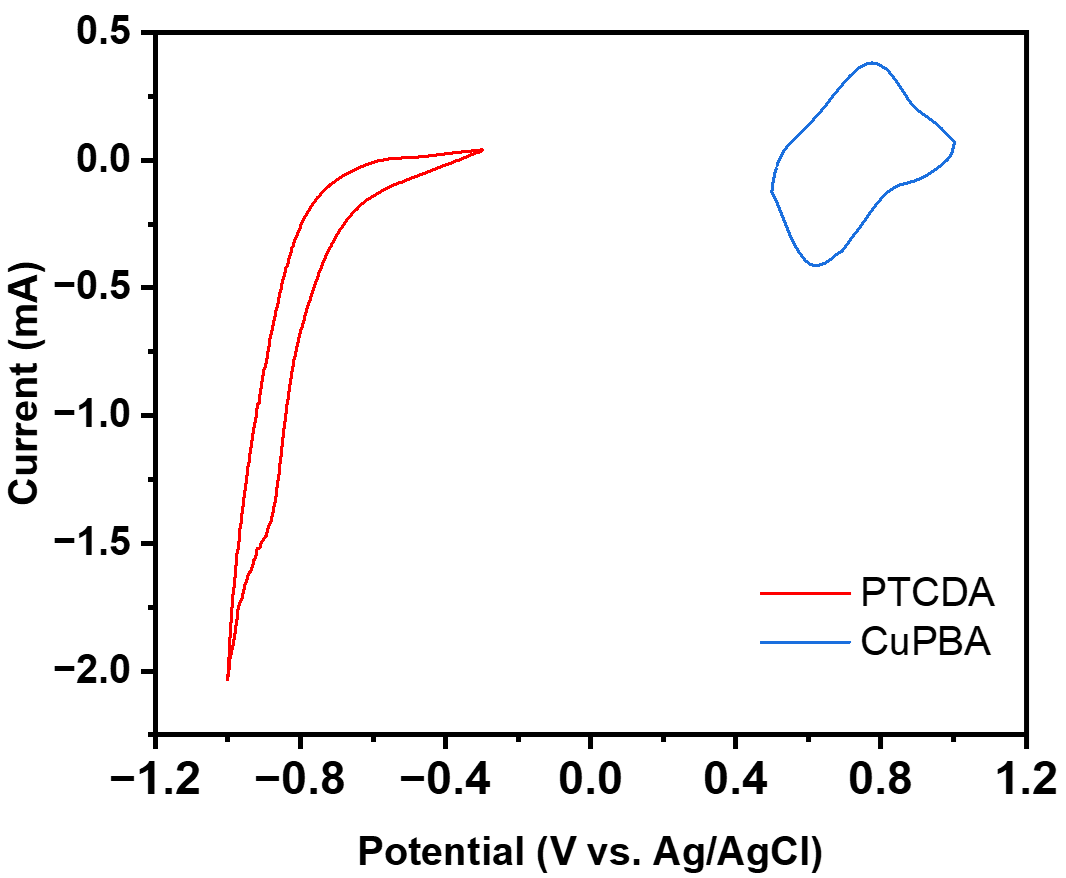


**Figure S15. CV curves of both PTCDA and CuPBA electrodes. Pt foil, Ag/AgCl, and 1 M Ca(NO_3_)_2_ aqueous solution were used as counter electrode, reference electrode, and electrolyte, respectively.**

**
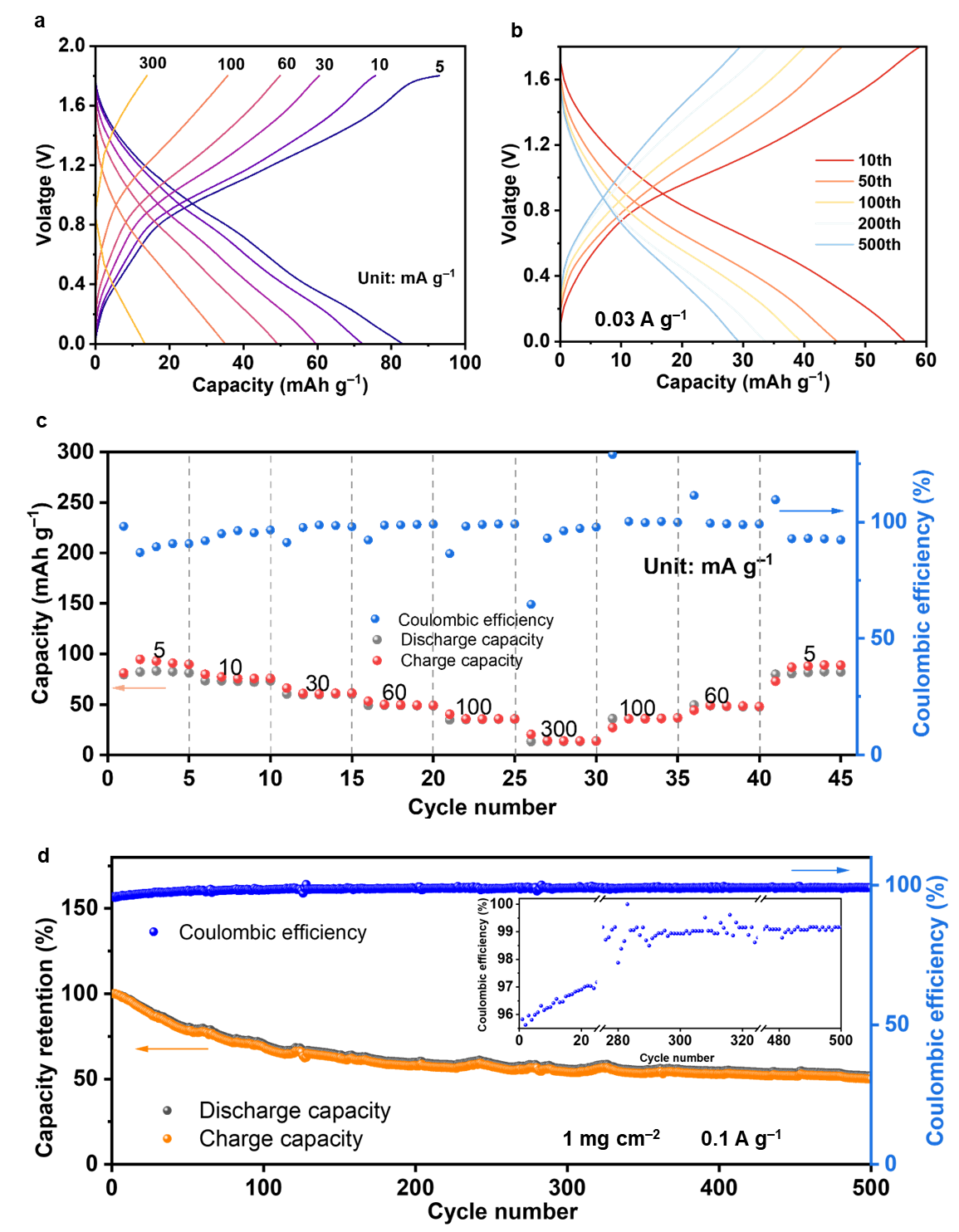
**

**Figure S16. Electrochemical performance of coin cell with PQ-COFs-QSSEs. a,** Charge/discharge curves at different current densities. **b,** Selected charge/discharge curves at 10^th^, 50^th^, 100^th^, 200^th^, and 500^th^ cycles at 0.03 A g^–1^. **c,** Charge/discharge capacities upon changing rates and their corresponding Coulombic efficiency. **d,** Long-term operation of the coin cell at 0.1 A g^–1^. Inset shows the coulombic efficiency at different cycles in detailed y-axis scale.

**Table S5. Repeated data for PTCDA|PT-COFs-QSSEs|CuPBA coin cells at 1 C.**

| **Samples** | **Discharge capacity (mAh g^–1^ )** | **Coulombic efficiency** |
| --- | --- | --- |
| Coin cell_1 | 155.0 | 98.7% |
| Coin cell_2 | 155.9 | 98.3% |
| Coin cell_3 | 151.2 | 99.1% |

**Table S6. Repeated data for PTCDA|PQ-COFs-QSSEs|CuPBA coin cells at 0.03 A g^–1^.**

| **Samples** | **Discharge capacity (mAh g^–1^ )** | **Coulombic efficiency** |
| --- | --- | --- |
| Coin cell_1 | 59.5 | 98.5% |
| Coin cell_2 | 60.2 | 98.4% |
| Coin cell_3 | 66.1 | 91.7% |

**Table S7. Comparison of liquid and quasi-solid-state calcium ion batteries performance in reported literature.**

| **Electrolyte** | **Anode** | **Cathode** | **Capacity** | **Stability** | **Ref.** |
| --- | --- | --- | --- | --- | --- |
| 0.8 M Ca(PF_6_)_2_ in 2:2:3:3 (v/v/v/v) EC/PC/DMC/EMC | Ca_x_M_y_ alloy | Graphite | 72 mAh g^–1^ at 0.1 A g^–1^ | 95% capacity retention at 0.1 A/g after 350 cycles | ^11^ |
| 2.5 M Ca(NO_3_)_2_ aqueous solution | PNDIE | CuHCF | 183 mAh g^–1^ at 1 C | 88% capacity retention at 10 C after 1,000 cycles | ^12^ |
| 3.4 M CaCl_2_ aqueous electrolyte | TB-COF | Ca_x_CuHCF | 253 mAh g^–1^ at 1 A g^–1^ | 0.01% capacity decay per cycle at 5 A/g after 3,000 cycles | ^13^ |
| 6.67 M CaCl_2_ aqueous solution | PTHAT-COF | MnPBA | 152.3 mAh g^–1^ at 1 A g^–1^ | 89.9% capacity retention at 20 A/g after 10,000 cycles | ^14^ |
| 1 M CaCl_2_ | HqTp | Active carbon | 119.5 mAh g^–1^ at 1 A g^–1^ | 73.7% capacity retention at 3 A/g after 1,600 cycles | ^15^ |
| 3.5 M Ca(FSI)_2_ in 2:2:3:3 (v/v/v/v) EC/PC/DMC/EMC | PTCDA | Graphite | 75.4 mAh g^–1^ at 0.1 A g^–1^ | 84.7% capacity retention at 0.1 A/g over 350 cycles | ^16^ |
| IL-GEs with 0.2 M Ca(TFSI)_2_ | V_2_O_5_ | Ca_3_Co_4_O_9_ | 140 mAh g^–1^ at 40 μA | 20 mAh/g, after 20 cycles | ^17^ |
| Ca(TFSI)_2_ dissolved in quaternary ester carbonates. | Active carbon | Mg_0.25_V_2_O_5_·H_2_O | 120 mAh g^–1^ at 0.02 A g^–1^ | 86.9% capacity retention at 0.1 A/g after 500 cycles | ^18^ |
| 0.7 M Ca(PF_6_)_2_ in EC/DMC/EMC (4:3:2 v/v/v). | Mesocarbon microbeads | Graphite | 66 mAh g^–1^ at 2 C | 94% capacity retention at 1 C after 300 cycles | ^19^ |
| 1 M CaCl_2_·2H_2_O aqueous electrolyte | PTCDI | CuPBA | 150 mAh g^–1^ at 0.1 A g^–1^ | 87.2% capacity retention at 1 A/g after 200 cycles | ^7^ |
| 0.25 M Ca[B(hfip)_4_]_2_ in DME | Calcium-tin alloy | 1,4-poly-  anthraquinone | 250 mAh g^–1^ at 0.26 A g^–1^ | 31.2% capacity retention at 1 C after 5,000 cycles | ^20^ |
| **PT-COFs-QSSEs** | **PTCDA** | **CuPBA** | **155 mAh g^–1^ at 0.15 A g^–1^** | **73% capacity retention at 1 A/g after 1,000 cycles** | **This work** |
| **PT-COFs-QSSEs** | **PTCDA** | **CuPBA** | **180 mAh g^–1^ at 0.1 A g^–1^** | **91% capacity retention at 0.1 A/g after 600 cycles** | **This work** |


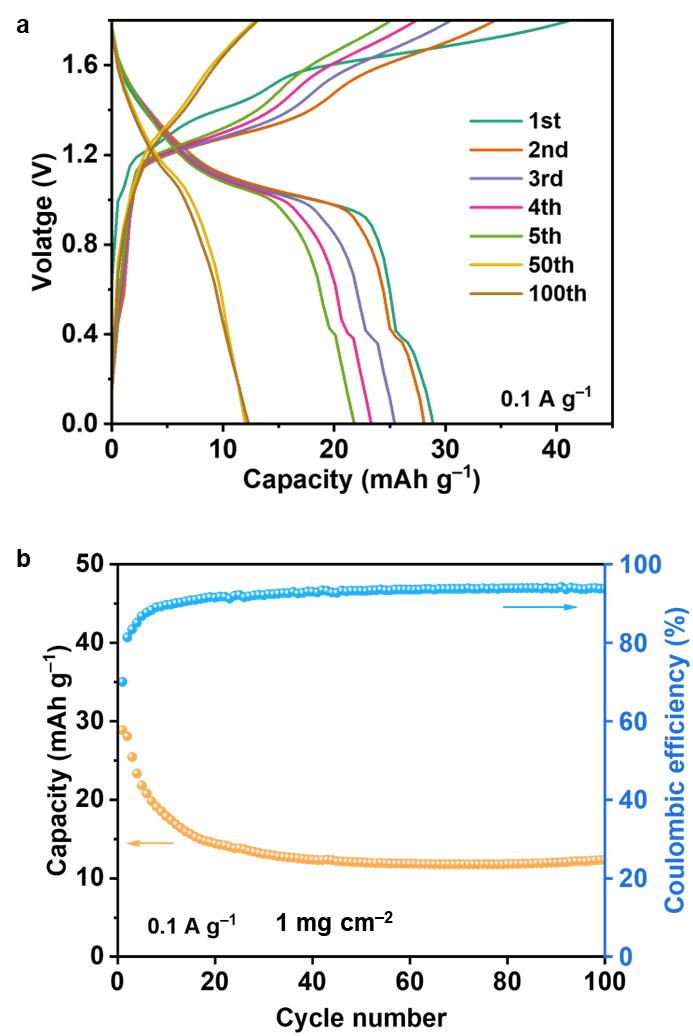


**Figure S17. Full cell with liquid electrolytes, PTCDA|0.5 M Ca(TFSI)_2_ in PC|CuPBA tested at 0.1 A g^–1^. a,** Selected charge/discharge curves up to 100 cycles. **b,** Discharge capacity and Coulombic efficiency of the cell up to 100 cycles.


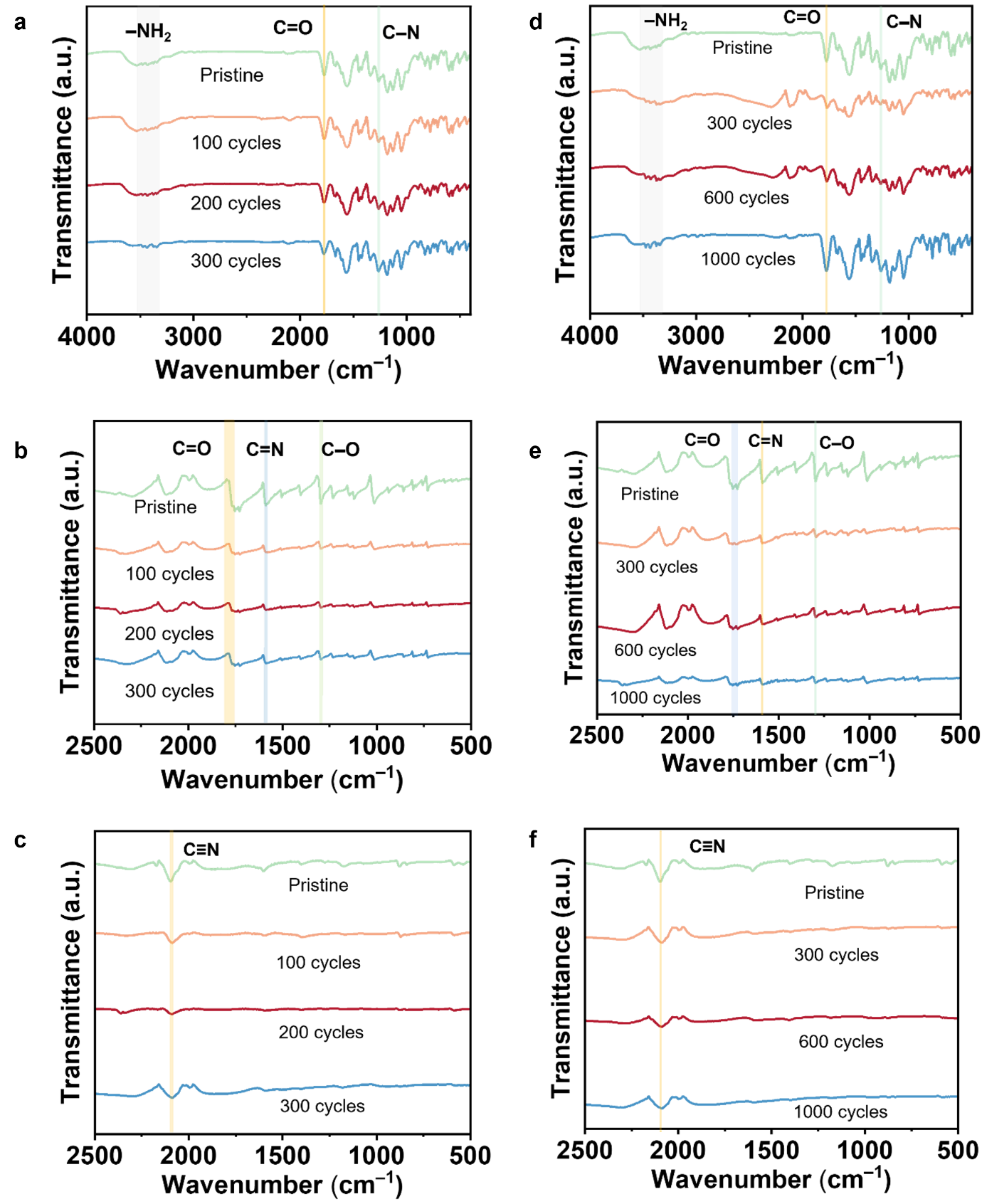


**Figure S18. *Ex situ* FTIR spectrum of PT-COFs-QSSEs, anode, and cathode after cycling. a–c,** PT-COFs-QSSEs, anode, cathode after different cycles at a current density of 0.1 A g^–1^. **d–f,** PT-COFs-QSSEs, anode, cathode after different cycles at a current density of 1.0 A g^–1^.


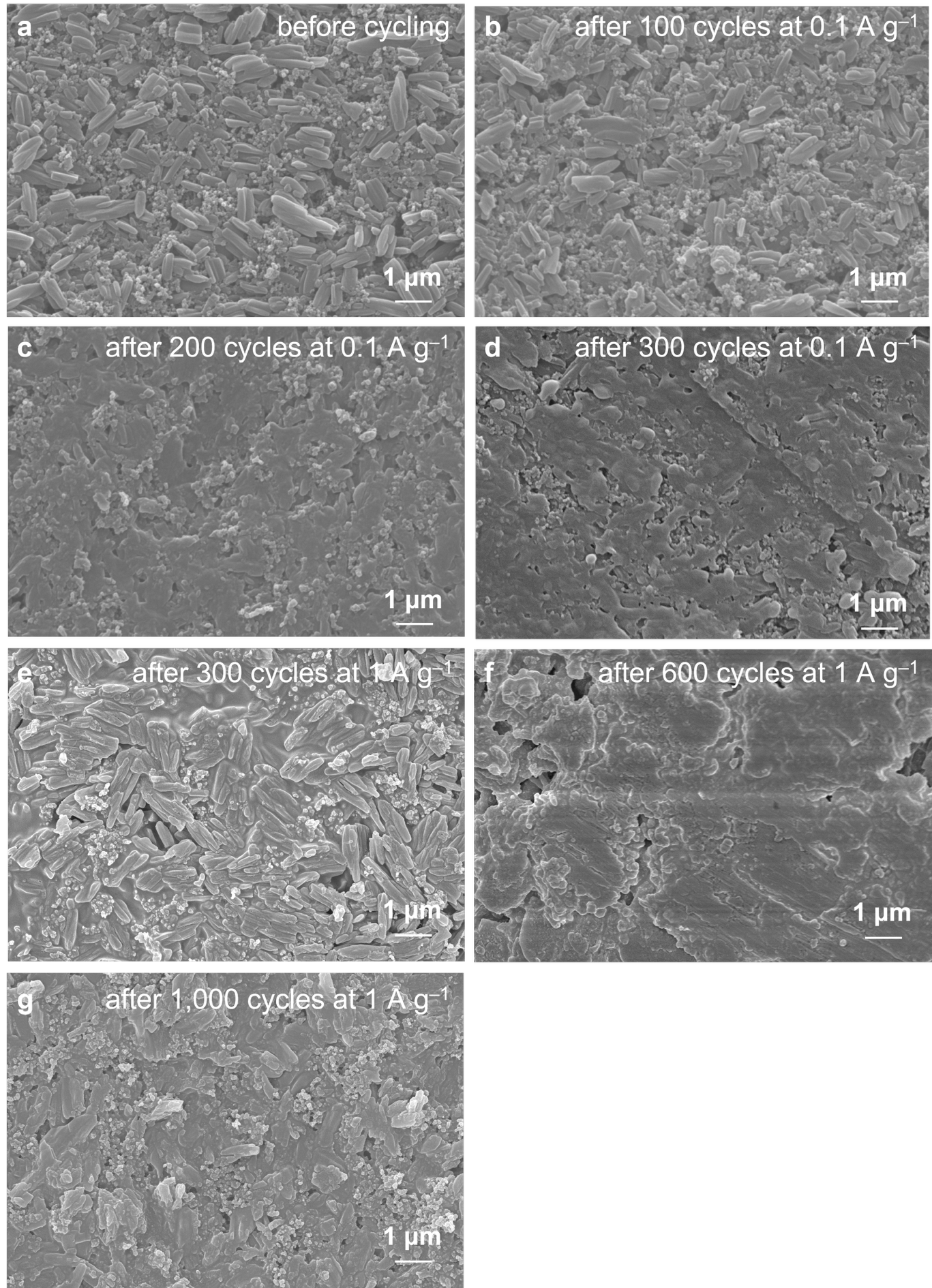


**Figure S19. SEM images of PTCDA anode surface morphologies of PTCDA|PT-COFs-QSSEs|CuPBA coin cells. a,** Before cycling. **b–d,** After 100, 200, and 300 cycles at a current density of 0.1 A g^–1^. **e–g,** After 300, 600, and 1,000 cycles at a current density of 1 A g^–1^.


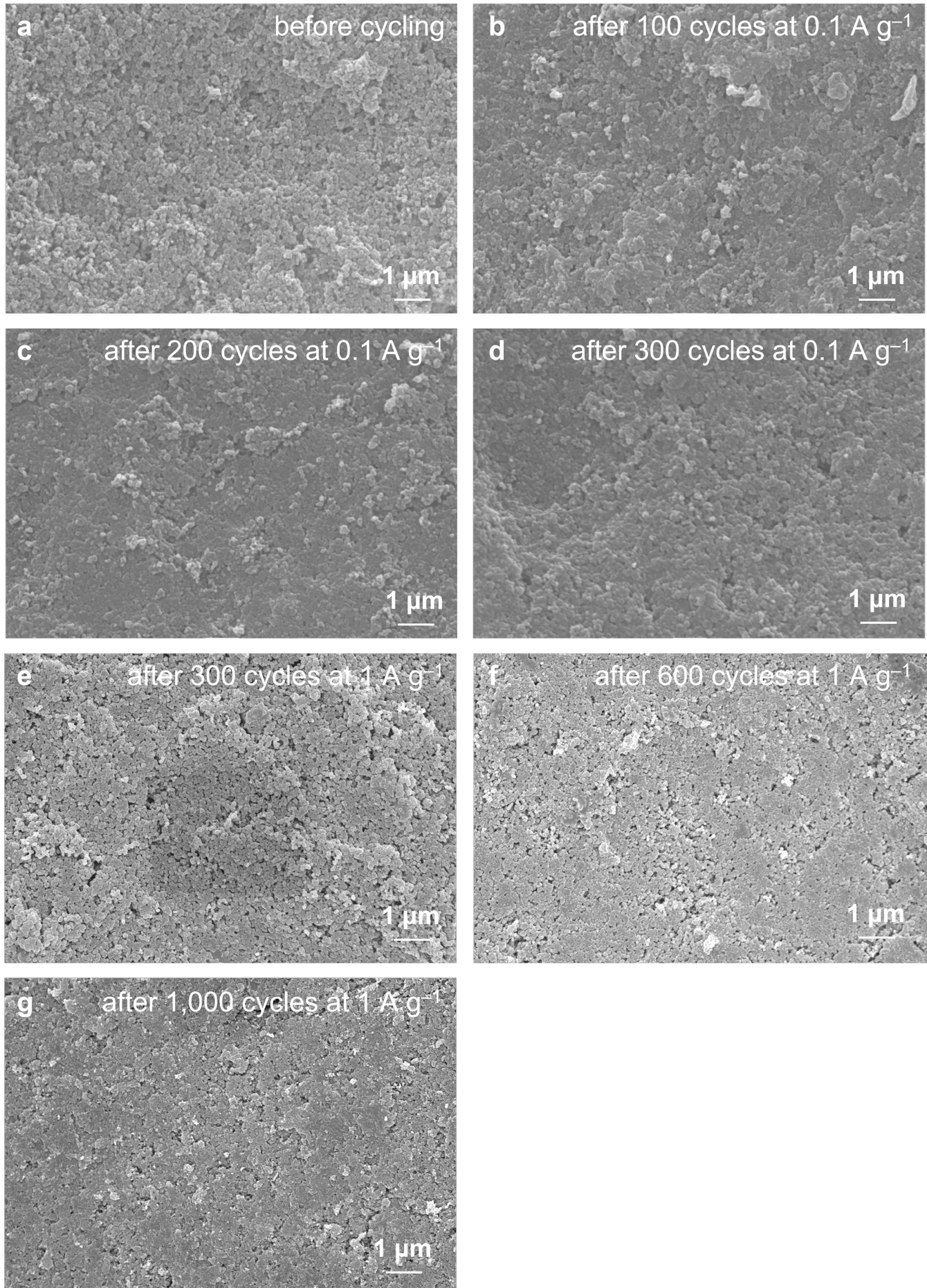


**Figure S20. SEM images of CuPBA** **cathode surface morphologies of PTCDA|PT-COFs-QSSEs|CuPBA coin cells. a,** Before cycling. **b–d,** After 100, 200, and 300 cycles at a current density of 0.1 A g^–1^. **e–g,** After 300, 600, and 1,000 cycles at a current density of 1 A g^–1^.


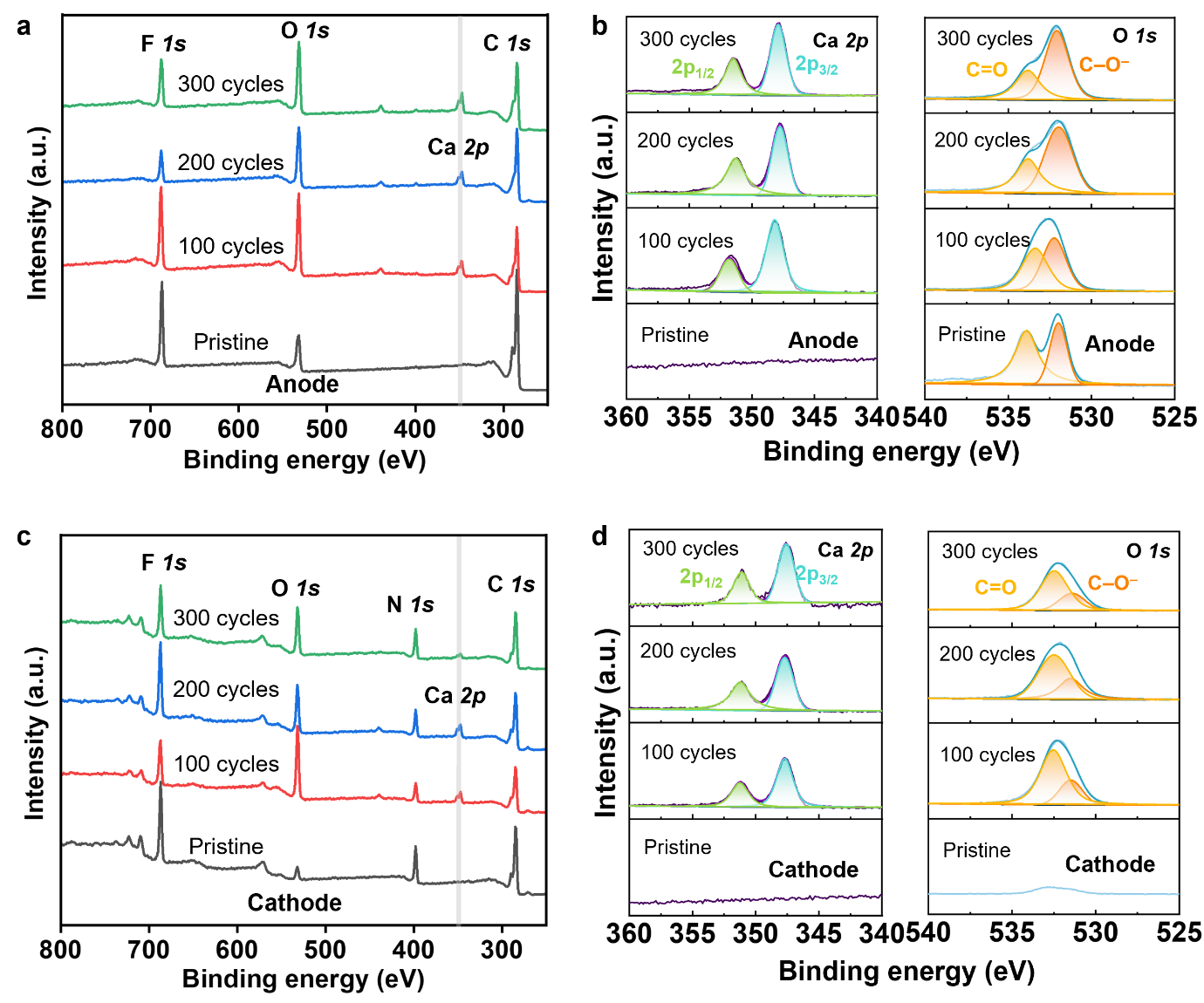


**Figure S21. XPS spectra of anode and cathode of PTCDA|PT-COFs-QSSEs|CuPBA coin cell before and after battery cycling at 0.1 A g^–1^. a, b,** anode, and its Ca *2p* and O *1s* peaks, respectively. **c, d,** cathode, and its Ca *2p* and O *1s* peaks, respectively.


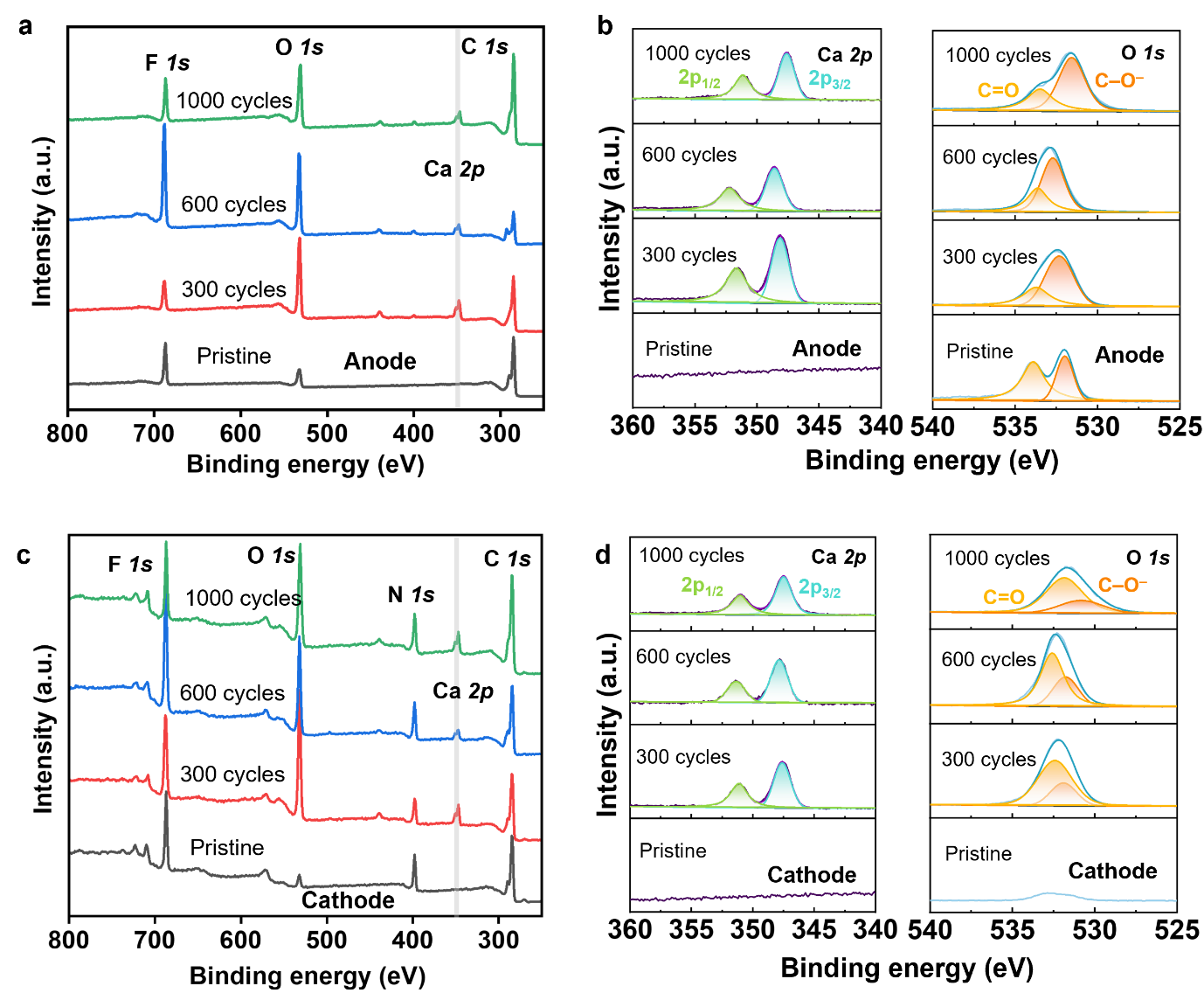


**Figure S22. XPS spectra of anode and cathode of PTCDA|PT-COFs-QSSEs|CuPBA coin cell before and after battery cycling at 1 A g^–1^. a, b,** anode, and its Ca *2p* and O *1s* peaks, respectively. **c, d,** cathode, and its Ca *2p* and O *1s* peaks, respectively.


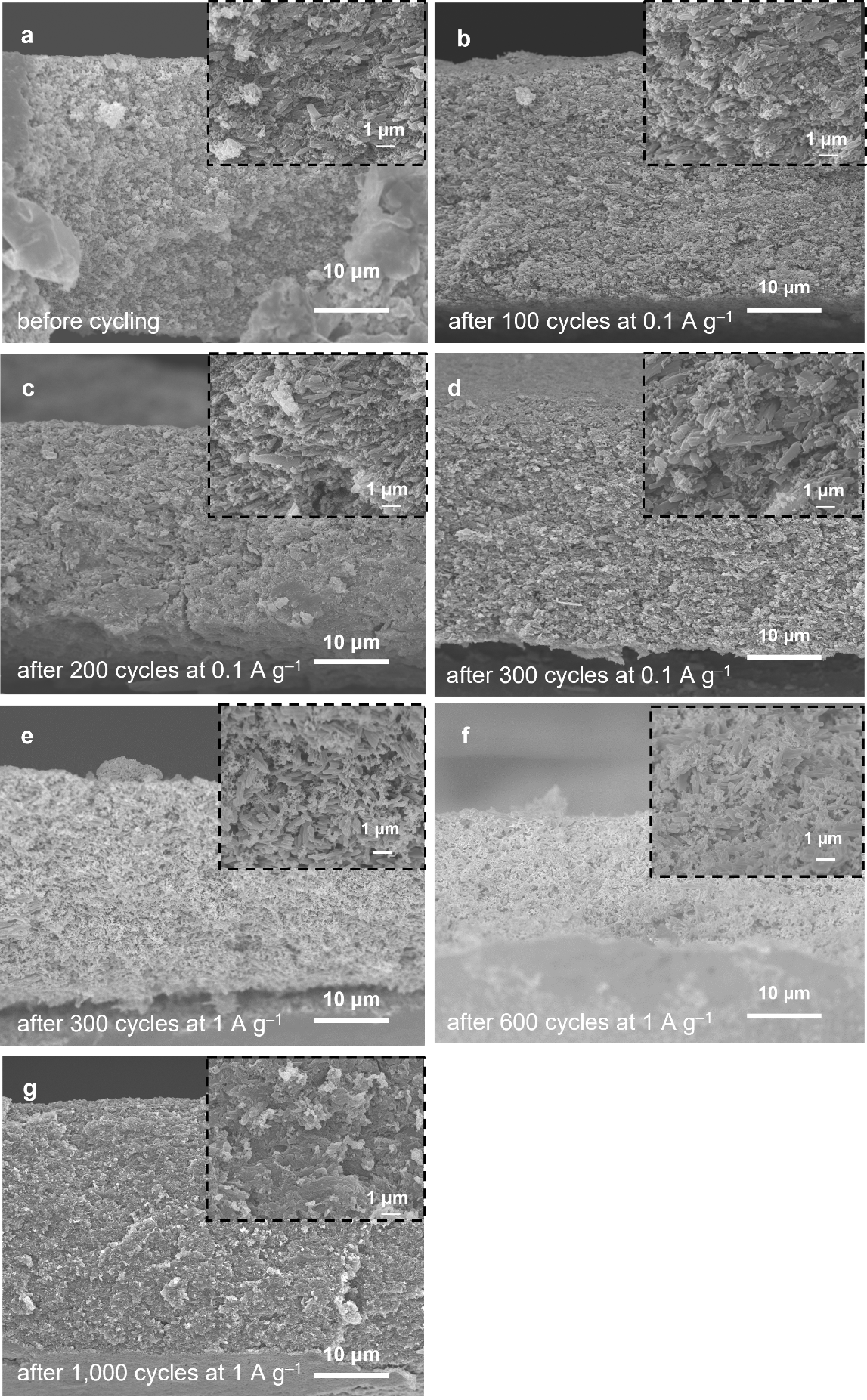


**Figure S23. SEM images of PTCDA anode cross-sectional morphologies of PTCDA|PT-COFs-QSSEs|CuPBA coin cells. a,** Before cycling. **b–d,** After 100, 200, and 300 cycles at current density of 0.1 A g^–1^. **e–g,** After 300, 600, and 1,000 cycles at current density of 1 A g^–1^. Insets are zoomed-in images.


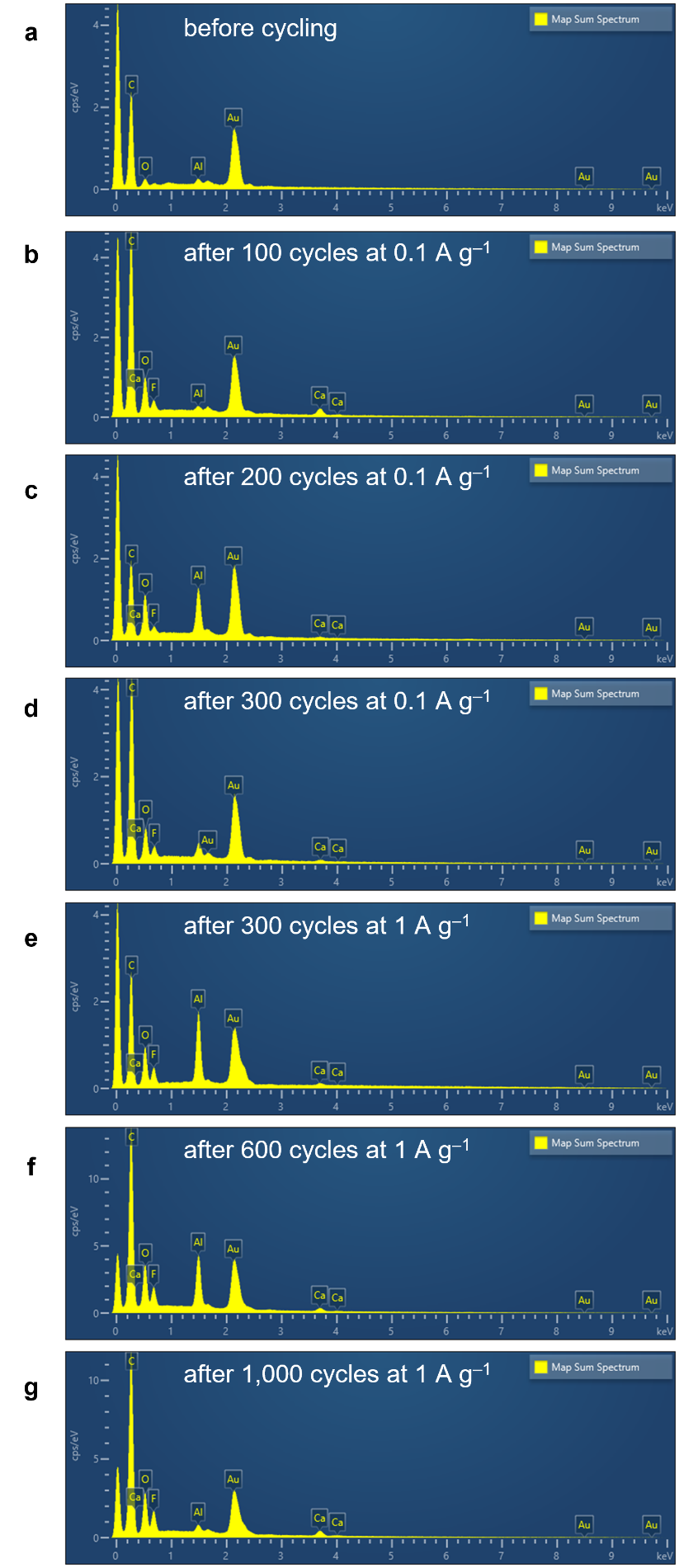


**Figure S24. Energy-dispersive spectroscopy (EDS) mapping spectrum** **of PTCDA** **anode cross-section of PTCDA|PT-COFs-QSSEs|CuPBA coin cell. a,** Before cycling. **b–d,** After 100, 200, and 300 cycles at current density of 0.1 A g^–1^. **e–g,** After 300, 600, and 1,000 cycles at current density of 1 A g^–1^.

**Table S8.** **Element atomic percentage in anode cross-section before and after cycling.**

| **Element** | **Atomic percentage (%)** | | | | | | |
| --- | --- | --- | --- | --- | --- | --- | --- |
|  |  | **0.1 A g^–1^** | | | **1 A g^–1^** | | |
|  | **Pristine** | **100 cycles** | **200 cycles** | **300 cycles** | **300 cycles** | **600 cycles** | **1000 cycles** |
| C | 87.97 | 81.31 | 68.81 | 84.01 | 71.14 | 75.18 | 74.48 |
| O | 5.01 | 12.79 | 20.27 | 10.87 | 17.12 | 15.85 | 17.41 |
| F | 2.63 | 2.63 | 2.66 | 2.61 | 5.30 | 5.03 | 6.62 |
| Ca | 0 | 0.98 | 0.26 | 0.23 | 0.13 | 0.50 | 0.95 |
| Al | 0.60 | 0.27 | 4.12 | 0 | 6.00 | 3.44 | 0.55 |
| Au | 3.79 | 2.02 | 3.88 | 2.28 | 0 | 0 | 0 |
| Total | 100 | 100 | 100 | 100 | 100 | 100 | 100 |


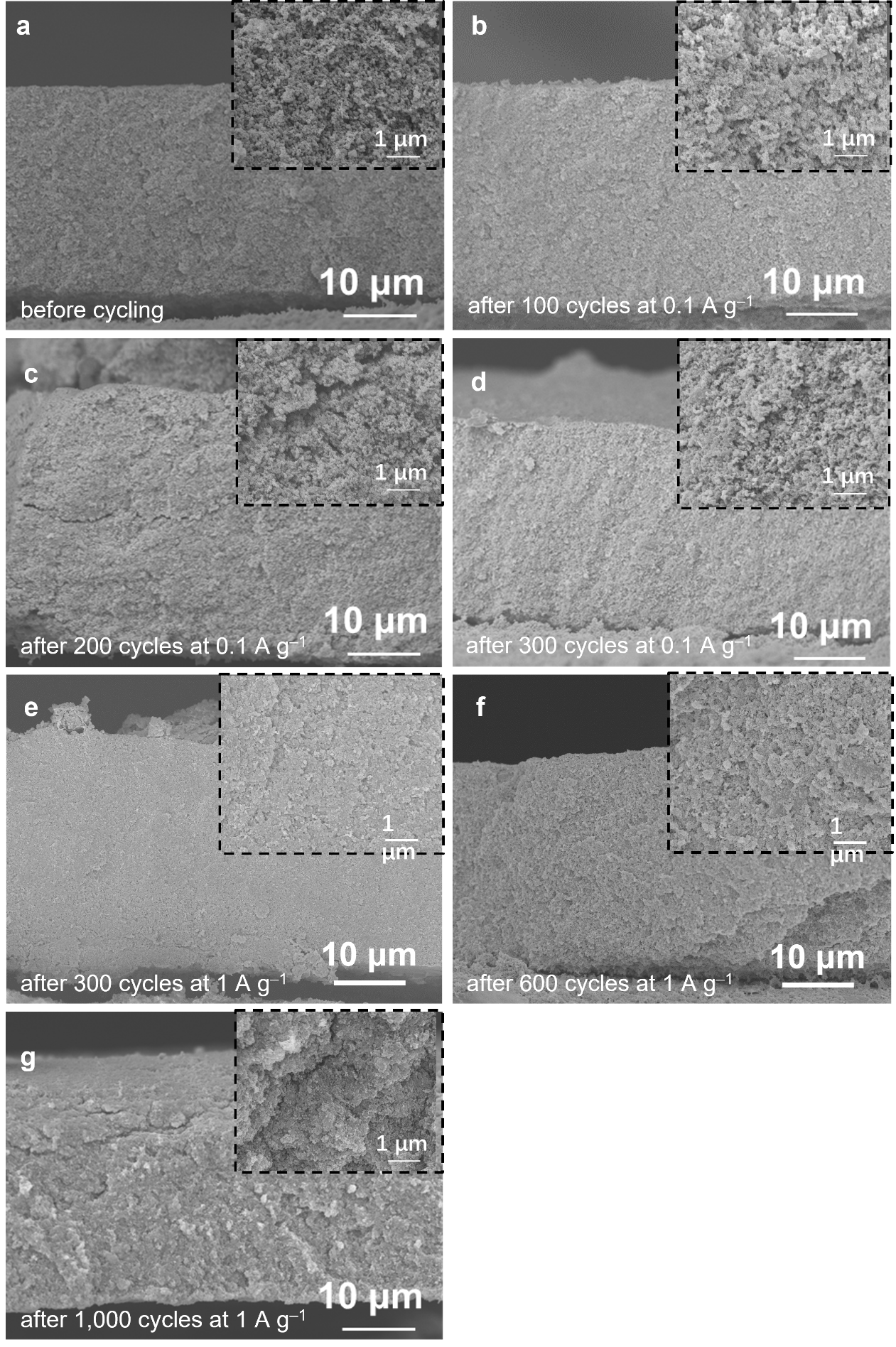


**Figure S25. SEM images of CuPBA cathode cross-sectional morphologies of PTCDA|PT-COFs-QSSEs|CuPBA coin cells. a,** Before cycling. **b–d,** After 100, 200, and 300 cycles at current density of 0.1 A g^–1^. **e–g,** After 300, 600, and 1,000 cycles at current density of 1 A g^–1^. Insets are zoomed-in images.


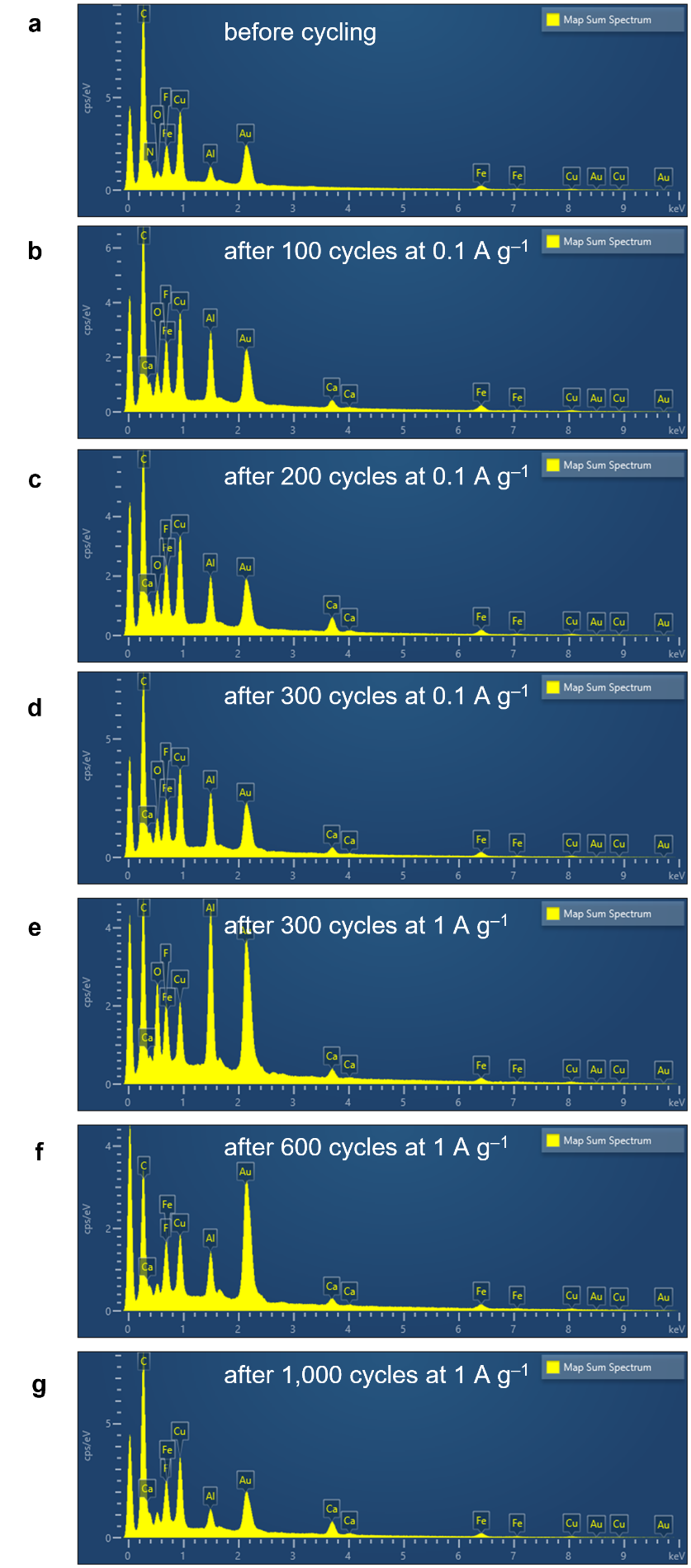


**Figure S26. EDS mapping spectrum** **of CuPBA cathode cross-section of PTCDA|PT-COFs-QSSEs|CuPBA coin cell. a,** Before cycling. **b–d,** After 100, 200, and 300 cycles at current density of 0.1 A g^–1^. **e–g,** After 300, 600, and 1,000 cycles at current density of 1 A g^–1^.

**Table S9.** **Element atomic percentage in cathode cross-section before and after cycling.**

| **Element** | **Atomic percentage (%)** | | | | | | |
| --- | --- | --- | --- | --- | --- | --- | --- |
|  |  | **0.1 A g^–1^** | | | **1 A g^–1^** | | |
|  | **Pristine** | **100 cycles** | **200 cycles** | **300**  **cycles** | **300 cycles** | **600 cycles** | **1000 cycles** |
| C | 84.29 | 71.80 | 72.97 | 74.24 | 73.15 | 78.14 | 82.42 |
| O | 3.76 | 7.70 | 6.77 | 7.56 | 5.91 | 0 | 0 |
| F | 2.50 | 5.44 | 5.51 | 4.41 | 6.30 | 8.42 | 5.03 |
| Ca | 0 | 1.65 | 0.87 | 0.76 | 0.88 | 1.13 | 1.82 |
| Al | 0.84 | 2.62 | 3.75 | 3.16 | 7.09 | 3.54 | 1.32 |
| Fe | 4.63 | 5.58 | 5.17 | 5.14 | 3.68 | 3.83 | 4.55 |
| Cu | 3.98 | 5.22 | 4.95 | 4.73 | 3.00 | 4.94 | 4.85 |
| Total | 100 | 100 | 100 | 100 | 100 | 100 | 100 |

**References**

1. Gao, H.; Neale, A. R.; Zhu, Q.; Bahri, M.; Wang, X.; Yang, H.; Xu, Y.; Clowes, R.; Browning, N. D.; Little, M. A.; Hardwick, L. J.; Cooper, A. I., A Pyrene-4,5,9,10-Tetraone-Based Covalent Organic Framework Delivers High Specific Capacity as a Li-Ion Positive Electrode. *J. Am. Chem. Soc.* **2022,** *144* (21), 9434-9442.

2. Li, M.; Liu, J.; Li, Y.; Xing, G.; Yu, X.; Peng, C.; Chen, L., Skeleton Engineering of Isostructural 2D Covalent Organic Frameworks: Orthoquinone Redox-Active Sites Enhanced Energy Storage. *CCS Chem.* **2021,** *3* (2), 696-706.

3. Xu, Y.; Cai, P.; Chen, K.; Chen, Q.; Wen, Z.; Chen, L., Hybrid Acid/alkali All Covalent Organic Frameworks Battery. *Angew. Chem. Int. Ed.* **2023,** *62* (18), e202215584.

4. Gao, H.; Zhu, Q.; Neale, A. R.; Bahri, M.; Wang, X.; Yang, H.; Liu, L.; Clowes, R.; Browning, N. D.; Sprick, R. S.; Little, M. A.; Hardwick, L. J.; Cooper, A. I., Integrated Covalent Organic Framework/Carbon Nanotube Composite as Li‐Ion Positive Electrode with Ultra‐High Rate Performance. *Adv. Energy Mater.* **2021,** *11* (39), 2101880.

5. Bravo, V.; Gil, S.; Costero, A. M.; Kneeteman, M. N.; Llaosa, U.; Mancini, P. M. E.; Ochando, L. E.; Parra, M., A new phenanthrene-based bis-oxime chemosensor for Fe(III) and Cr(III) discrimination. *Tetrahedron* **2012,** *68* (24), 4882-4887.

6. Evans, J.; Vincent, C. A.; Bruce, P. G., Electrochemical measurement of transference numbers in polymer electrolytes. *Polymer* **1987,** *28*, 2324-2328.

7. Li, R.; Yu, J.; Chen, F.; Su, Y.; Chan, K. C.; Xu, Z. L., High‐Power and Ultrastable Aqueous Calcium‐Ion Batteries Enabled by Small Organic Molecular Crystal Anodes. *Adv. Funct. Mater.* **2023,** *33* (30), 2214304.

8. Peng, J.; Zhang, W.; Liu, Q.; Wang, J.; Chou, S.; Liu, H.; Dou, S., Prussian Blue Analogues for Sodium-Ion Batteries: Past, Present, and Future. *Adv. Mater.* **2022,** *34* (15), e2108384.

9. Frisch, M. J.; Trucks, G. W.; Schlegel, H. B.; Scuseria, G. E.; Robb, M. A.; Cheeseman, J. R.; Scalmani, G.; Barone, V.; Petersson, G. A.; Nakatsuji, H.; Li, X.; Caricato, M.; Marenich, A. V.; Bloino, J.; Janesko, B. G.; Gomperts, R.; Mennucci, B.; Hratchian, H. P.; Ortiz, J. V.; Izmaylov, A. F.; Sonnenberg, J. L.; Williams; Ding, F.; Lipparini, F.; Egidi, F.; Goings, J.; Peng, B.; Petrone, A.; Henderson, T.; Ranasinghe, D.; Zakrzewski, V. G.; Gao, J.; Rega, N.; Zheng, G.; Liang, W.; Hada, M.; Ehara, M.; Toyota, K.; Fukuda, R.; Hasegawa, J.; Ishida, M.; Nakajima, T.; Honda, Y.; Kitao, O.; Nakai, H.; Vreven, T.; Throssell, K.; Montgomery Jr., J. A.; Peralta, J. E.; Ogliaro, F.; Bearpark, M. J.; Heyd, J. J.; Brothers, E. N.; Kudin, K. N.; Staroverov, V. N.; Keith, T. A.; Kobayashi, R.; Normand, J.; Raghavachari, K.; Rendell, A. P.; Burant, J. C.; Iyengar, S. S.; Tomasi, J.; Cossi, M.; Millam, J. M.; Klene, M.; Adamo, C.; Cammi, R.; Ochterski, J. W.; Martin, R. L.; Morokuma, K.; Farkas, O.; Foresman, J. B.; Fox, D. J. *Gaussian 16 Rev. B.01*, Wallingford, CT, 2016.

10. GaussView, Version 6, Roy Dennington, Todd A. Keith, and John M. Millam. *Semichem Inc., Shawnee Mission, KS,* **2016**.

11. Wang, M.; Jiang, C.; Zhang, S.; Song, X.; Tang, Y.; Cheng, H. M., Reversible calcium alloying enables a practical room-temperature rechargeable calcium-ion battery with a high discharge voltage. *Nat. Chem.* **2018,** *10* (6), 667-672.

12. Gheytani, S.; Liang, Y.; Wu, F.; Jing, Y.; Dong, H.; Rao, K. K.; Chi, X.; Fang, F.; Yao, Y., An Aqueous Ca-Ion Battery. *Adv. Sci.* **2017,** *4* (12), 1700465.

13. Zhang, S.; Zhu, Y. L.; Ren, S.; Li, C.; Chen, X. B.; Li, Z.; Han, Y.; Shi, Z.; Feng, S., Covalent Organic Framework with Multiple Redox Active Sites for High-Performance Aqueous Calcium Ion Batteries. *J. Am. Chem. Soc.* **2023,** *145* (31), 17309-17320.

14. Wang, C.; Li, R.; Zhu, Y.; Wang, Y.; Lin, Y.; Zhong, L.; Chen, H.; Tang, Z.; Li, H.; Liu, F.; Zhi, C.; Lv, H., A Pyrazine‐Pyridinamine Covalent Organic Framework as a Low Potential Anode for Highly Durable Aqueous Calcium‐Ion Batteries. *Adv. Energy Mater.* **2023,** *14* (1), 2302495.

15. Li, L.; Zhang, G.; Deng, X.; Hao, J.; Zhao, X.; Li, H.; Han, C.; Li, B., A covalent organic framework for high-rate aqueous calcium-ion batteries. *J Mater Chem A* **2022,** *10* (39), 20827-20836.

16. Li, J.; Han, C.; Ou, X.; Tang, Y., Concentrated Electrolyte for High-Performance Ca-Ion Battery Based on Organic Anode and Graphite Cathode. *Angew. Chem. Int. Ed.* **2022,** *61* (14), e202116668.

17. Biria, S.; Pathreeker, S.; Genier, F. S.; Hosein, I. D., A Highly Conductive and Thermally Stable Ionic Liquid Gel Electrolyte for Calcium-Ion Batteries. *ACS Appl. Polym. Mater.* **2020,** *2* (6), 2111-2118.

18. Xu, X.; Duan, M.; Yue, Y.; Li, Q.; Zhang, X.; Wu, L.; Wu, P.; Song, B.; Mai, L., Bilayered Mg0.25V2O5·H2O as a Stable Cathode for Rechargeable Ca-Ion Batteries. *ACS Energy Lett.* **2019,** *4* (6), 1328-1335.

19. Wu, S.; Zhang, F.; Tang, Y., A Novel Calcium-Ion Battery Based on Dual-Carbon Configuration with High Working Voltage and Long Cycling Life. *Adv. Sci.* **2018,** *5* (8), 1701082.

20. Zhao-Karger, Z.; Xiu, Y. L.; Li, Z. Y.; Reupert, A.; Smok, T.; Fichtner, M., Calcium-tin alloys as anodes for rechargeable non-aqueous calcium-ion batteries at room temperature (vol 13, 3849, 2022). *Nat. Commun.* **2022,** *13* (1), 3849.
